# Modelling of primary ciliary dyskinesia using patient‐derived airway organoids

**DOI:** 10.15252/embr.202052058

**Published:** 2021-10-25

**Authors:** Jelte van der Vaart, Lena Böttinger, Maarten H Geurts, Willine J van de Wetering, Kèvin Knoops, Norman Sachs, Harry Begthel, Jeroen Korving, Carmen Lopez‐Iglesias, Peter J Peters, Kerem Eitan, Alex Gileles‐Hillel, Hans Clevers

**Affiliations:** ^1^ Hubrecht Institute, Royal Netherlands Academy of Arts and Sciences (KNAW) University Medical Centre Utrecht Utrecht The Netherlands; ^2^ Oncode Institute Hubrecht Institute Utrecht The Netherlands; ^3^ The Maastricht Multimodal Molecular Imaging Institute Maastricht University Maastricht The Netherlands; ^4^ Division of Cell Biology, Immunology and Cancer Research Hebrew University‐Hadassah Medical School Jerusalem Israel; ^5^ Department of Paediatrics, Paediatric Pulmonology and Sleep Hadassah Hebrew University Medical Centre Jerusalem Israel; ^6^ Present address: Vertex Inc San Diego CA USA

**Keywords:** airway organoids, ciliated cell, primary ciliary dyskinesia, pulmonary differentiation, Molecular Biology of Disease, Stem Cells & Regenerative Medicine

## Abstract

Patient‐derived human organoids can be used to model a variety of diseases. Recently, we described conditions for long‐term expansion of human airway organoids (AOs) directly from healthy individuals and patients. Here, we first optimize differentiation of AOs towards ciliated cells. After differentiation of the AOs towards ciliated cells, these can be studied for weeks. When returned to expansion conditions, the organoids readily resume their growth. We apply this condition to AOs established from nasal inferior turbinate brush samples of patients suffering from primary ciliary dyskinesia (PCD), a pulmonary disease caused by dysfunction of the motile cilia in the airways. Patient‐specific differences in ciliary beating are observed and are in agreement with the patients' genetic mutations. More detailed organoid ciliary phenotypes can thus be documented in addition to the standard diagnostic procedure. Additionally, using genetic editing tools, we show that a patient‐specific mutation can be repaired. This study demonstrates the utility of organoid technology for investigating hereditary airway diseases such as PCD.

## Graphical abstract

The differentiation of adult stem cell‐derived airway organoids towards ciliated cells is optimized, which allows for improved disease characterisation and genetic editing, demonstrating the utility of organoid technology for investigating hereditary airway diseases.

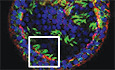

## Introduction

The human airways are continuously exposed to pathogens and other foreign material. Constitutive clearance is essential for maintaining the healthy state. To this end, a layer of mucus produced by club and goblet cells is transported towards the oral cavity by ciliated cells. Particles trapped in the layer of secreted mucus are thus removed from the body. Defects in this mucociliary clearance (MCC) mechanism lead to respiratory distress due to impaired oxygen transport, but also to frequent and/or chronic infections (Bustamante‐Marin & Ostrowski, 2017).

The important role of MCC is best demonstrated in primary ciliary dyskinesia (PCD), a rare genetic disorder, which leads to lifelong recurrent respiratory tract infections. PCD is a rare genetic disorder, which manifests itself in the dysfunction of the cilia, leading to reduced MCC capacity of the airways. While the incidence of PCD is approximately one in 15,000–30,000, this condition is likely underdiagnosed due to suboptimal diagnostic parameters (Leigh *et al*, 2011). Symptoms usually begin early in life and include chronic nasal discharge and wet cough, progressing in childhood to recurrent upper and lower airway infections and eventual bronchiectasis. As cilia are also present at the embryonic node, defects in nodal cilia may cause abnormalities of left–right laterality determination (situs abnormalities) in addition to male infertility (Mullowney *et al*, 2014; Goutaki *et al*, 2016; Knowles *et al*, 2016; Leigh *et al*, 2019; Lucas *et al*, 2020). In recent years, major advances have been made in the diagnosis and understanding of PCD, and large‐scale sequencing approaches have led to the discovery of new causal genes. So far, mutations in over 40 PCD causing genes have been identified. Ultrastructural ciliary defects are observed in only 70% of the clinically confirmed cases (Lucas *et al*, 2020). There remains a group of patients that cannot be diagnosed using standard methods.

Coordination of beating direction and frequency of cilia is essential for productive MCC. Motile cilia are evolutionarily conserved. These cilia are complex organelles that are carried by specialized cells of the respiratory tract, brain ventricles and reproductive organs. Disorders of motile cilia are referred commonly as motile ciliopathies, and PCD is the most common one. Genes mutated in PCD encode ultrastructural components of the cilia axoneme, or components of cytoplasmic complexes required for preassembly of the structural elements. Contrary to what is traditionally thought, mutations causing PCD do not necessarily affect the ultrastructural composition of the cilia axoneme and may only have a (subtle) effect on ciliary movement. No single test can confirm a diagnosis of motile ciliopathy, which is based on a combination of tests including nasal nitric oxide measurement, transmission electron microscopy, immunofluorescence and genetic testing (Gileles‐Hillel *et al*, 2020), and high‐speed video microscopy to analyse ciliary beat frequency (CBF) and ciliary motion pattern (CMP) (Knowles *et al*, 2016; Leigh *et al*, 2019; Lucas *et al*, 2020). Lack of an adaptable, long‐term *in vitro* model hampers a more detailed study of ciliary dyskinesia in individual patients: patient‐derived primary airway cell cultures are usually only viable for a few weeks.

Other model systems which are currently used for modelling PCD are unicellular organisms and vertebrate animal models including zebrafish and xenopus. While these models allow for the modelling of some aspects of PCD, the models do not fully recapitulate human disease (Poprzeczko *et al*, 2019). Similarly, genetic mouse models present with PCD‐like phenotypes when known PCD‐causing mutations are introduced. However, due to the extensive spectrum of PCD‐causing mutations and the underlying patient‐specific genome, the variety of PCD phenotypes cannot be modelled in mice (Norris & Grimes, 2012). This underlines the need for a patient‐specific model system to identify personalized disease phenotypes. Currently, patient cells are cultured in air–liquid interface (ALI) cultures which require substantial numbers of cells. More importantly, ALI cultures cannot be passaged over long periods of time and thereby provide limited time span for diagnostics and research (Hirst *et al*, 2010, 2014). The search for a patient‐specific long‐term model system therefore remains.

To date, therapeutic interventions can only alleviate PCD patients’ symptoms. Most patients suffer from a lifelong chronic respiratory morbidity, with varying severity of the symptoms. Yet, the disease progresses and ultimately may lead to respiratory insufficiency and even the need for lung transplantation. The heterogeneous nature of the disease, explained to some degree by the different ciliary genes involved, suggests the need for personalized therapeutic approaches. The recent development of human airway organoid (AO) culture has opened the possibility of studying airway diseases in patient‐specific primary material in models that can be manipulated and studied, while remaining genetically and phenotypically stable over long periods of time. These AOs can be generated from small biopsies and expanded over time while maintaining its potential to differentiate (Sachs *et al*, 2019; van der Vaart & Clevers, 2020). While 2D cultures can be generated from small biopsies, the expansion of these basal cells is limited for a few passages (van der Vaart & Clevers, 2020).

Furthermore, MCC and ciliary function are impaired in many chronic respiratory conditions such as COPD, bronchiectasis and chronic smoking. While efforts in generating AOs has resulted in limited ciliated cell numbers when spontaneous differentiation was achieved (Sachs *et al*, 2019), differentiation of ciliated cells in 3D model systems was achieved by using undefined commercially available medium (Zhou *et al*, 2018). Using current knowledge of ciliated cell differentiation could identify robust yet defined conditions that allow for ciliated cell differentiation in AOs. A defined ciliary organoid model will allow to develop drugs that may improve ciliary function, which are currently unavailable (Wallmeier *et al*, 2020). This study explores patient‐specific AO establishment from PCD patients and the characterization of their ciliary defect, with the aim of establishing long‐lived models of this disease.

## Results

**Figure 1:**
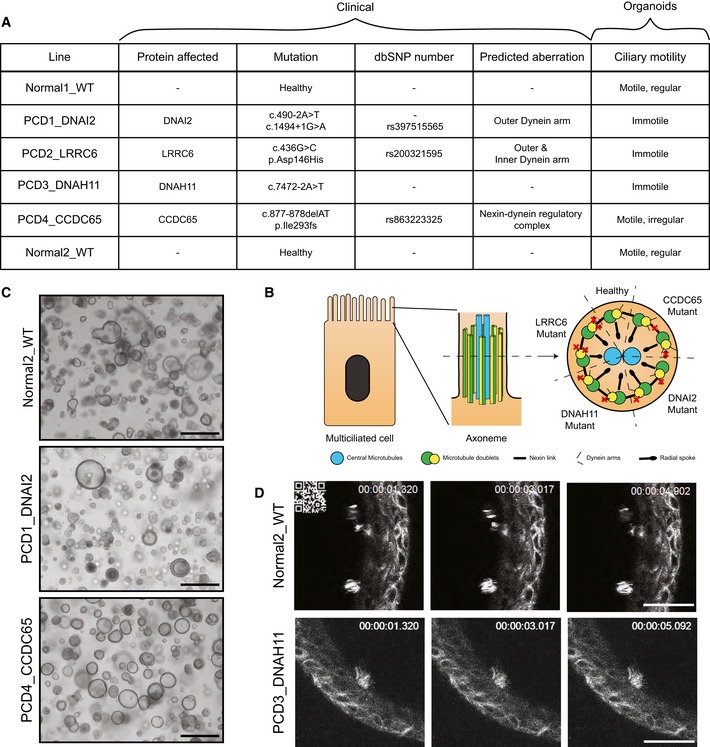
Generation of human airway organoids from primary cilia dyskinesia patients Summary/overview of the airway organoid lines used in this study. Details regarding the mutational status, affected protein, dbSNP number, cilia structural component predicted to be affected and ciliary motility are included. Genetic mutations of generated AOs were confirmed by Sanger sequencing (Appendix Fig S1).Schematic figure highlighting the cilia inner structure (axoneme). The cross section of the axoneme shows the genes affected in generated AOs and their predicted effect on the different structural components of the axoneme.Representative bright‐field images of PCD (PCD1_DNAI2 and PCD4_CCDC65) and healthy control AOs (Normal2_WT) displaying their morphological similarity. Scale bar = 500 µm.Representative montage of SiR‐tubulin live imaging (Movie EV1) showing the difference in cilia motility between healthy and PCD AOs. Healthy AOs (Normal2_WT) (upper panel) display normal cilia motility, whereas cilia in PCD AOs (PCD3_DNAH11) are immotile (lower panel). All organoids were cultured in AO medium. Time is indicated in seconds. Scale bar = 25 µm.

To establish patient‐specific PCD organoids, we obtained airway organoid (AO) cultures from nasal inferior turbinate brush (NITB) samples collected from healthy and affected individuals with known mutations in PCD‐related genes. Several studies have shown the use of nasal epithelium as model for airway diseases like PCD (Barlocco *et al*, 1991; Hirst *et al*, 2010; Lucas *et al*, 2014; Adil *et al*, 2017; Marthin *et al*, 2017; Shoemark *et al*, 2017; Bricmont *et al*, 2020; Coles *et al*, 2020). The non‐invasively received NITB samples were obtained from 4 patients and 2 healthy controls. NITB‐derived AOs have shown effectiveness as airway epithelium model (Liu *et al*, 2020; Anderson *et al*, 2021; Rijsbergen *et al*, 2021). Patients were diagnosed with PCD and their genomes sequenced. Clinical data from two of the patients were published previously (Horani, Brody, *et al*, 2013; Horani, Ferkol, *et al*, 2013). The patients carried mutations in *Dynein Axonemal Intermediate Chain 2* (*DNAI2*), *Leucine Rich Repeat Containing 6* (*LRRC6*), *Dynein Axonemal Heavy Chain 11* (*DNAH11*) and *Coiled‐Coil Domain Containing 65* (*CCDC65*) respectively (Fig 1A). *DNAI2* mutations are predicted to lead to dysfunctional outer dynein arms in PCD1_DNAI2. Homozygous amino acid alteration D146H in LRRC6 causes aberrations in both outer as inner dynein arms in PCD2_LRRC6. Deletions in CCDC65 lead to loss of microtubule‐coupling nexin links in PCD4_CCDC65 (Fig 1B). NITB samples of both nostrils were placed in tubes containing transport medium and transported on ice from Jerusalem to Utrecht. Within 24 h after collection, the samples were received and processed by mechanical and enzymatic digestion. Only limited numbers of cells were plated yet AOs formed which could be expanded exponentially, frozen down and thawed repeatedly. The majority of the established organoids were cystic and showed a comparable phenotype to the previously published AOs (Fig 1C) (Sachs *et al*, 2019). Percentage of cystic organoids varied between donors and passages within a single line; only cystic organoids contained visible ciliary cells. AOs could be maintained for > 35 passages (> 1 year). Patient mutations were verified in the established organoid lines (Appendix Fig S1A–E). While limited numbers of cells with beating cilia were observed in healthy AOs (Fig 1D, upper panel and Movie EV1a), immotile cilia were observed in 3 out of 4 patient‐derived AOs (Fig 1D, lower panel and Movie EV1b). This observation was confirmed using the standard method of mucociliary differentiation using ALI cultures (Hirst *et al*, 2010) (Movie EV2). Using high‐speed imaging techniques, cultures of healthy AOs showed regular CBF of cilia (Movie EV2a,b), while cultures of PCD1_DNAI2 displayed no visible beating in ALI cultures (Movie EV2c,d). Taken together, NITB‐derived AOs from known PCD patients appeared to maintain disease phenotype in culture, but limited numbers of ciliated cells in expansion conditions hampered robust PCD modelling.

**Figure 2:**
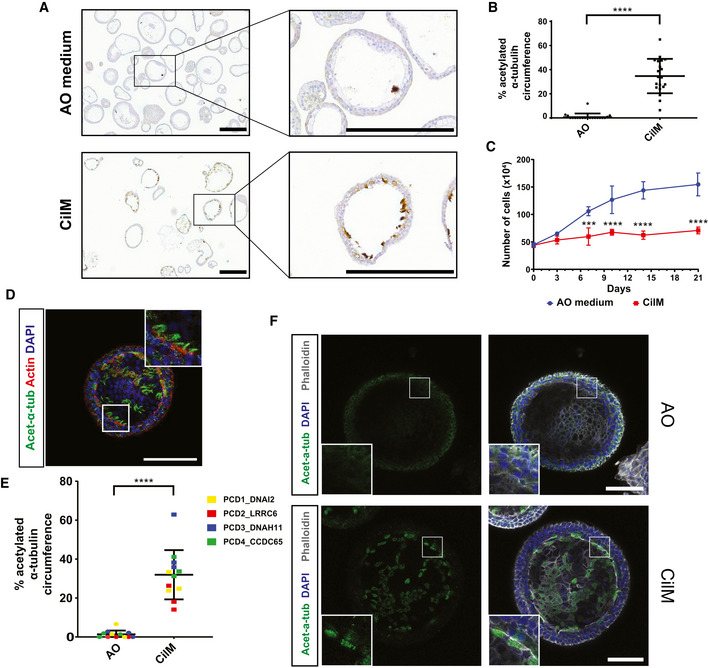
Cilia medium (CilM) promotes differentiation of ciliated cells in human airway organoids Representative immunohistochemistry images of healthy human AOs (Normal1_WT) stained for acetylated‐α‐tubulin. An increased number of ciliated cells were observed in organoids cultured in CilM (21 days) compared to AO medium. Scale bar = 200 µm.Quantification of acetylated‐α‐tubulin^+^ circumference as a measure of ciliated cell numbers shows an increase upon culturing healthy AOs in CilM (21 days) compared to AO. Each dot represents an analysed organoid. ****P < 0.0001 using Student *t*‐test. Bars = mean ± SD. *N* = 10.Quantification of cell numbers following culturing of organoids in CilM (red) or AO (blue) medium. Upon differentiation in CilM, organoids do no longer expand and stabilize in cell number while organoids in AO medium keep expanding seen by an increase in cell number. Organoids were first cultured in AO media for 14 days and were then either continued to be cultured in AO or subjected to CilM. The number of cells was quantified at each indicated time point following the first 14 days in AO media. Two donors were tested in triplicate. ***P < 0.001 and ****P < 0.0001 using Student *t*‐test. *N* = 3. Error bars = SD.Representative image of healthy AOs (Normal1_WT) in CilM (21 days) stained for cilia (acetylated‐α‐tubulin (Ac‐α‐tub)), cellular membrane (Actin) and nucleus (DAPI). Scale bar = 100 µm. Ciliated cells occur throughout the organoid as acetylated‐α‐tubulin (Ac‐α‐tub)^+^ cells.Quantification of acetylated‐α‐tubulin^+^ circumference as a measure of ciliated cell numbers shows an increase upon culturing PCD AOs in CilM (21 days) compared to AO. Each dot represents ten analysed organoids in a single differentiation experiment. Colour of the dot indicates donor line. ****P < 0.0001 using Student *t*‐test. *N* = 12. Bars = mean ± SD.Representative IF image of PCD AOs (PCD3_DNAH11) in CilM (14 days) stained for cilia (acetylated‐α‐tubulin (Ac‐α‐tub)), cellular membrane (Phalloidin) and nucleus (DAPI). Scale bar = 100 µm. Ciliated cells can be observed following culture of the organoids in CilM but not in AO medium. Images are similar to Fig EV3.

**Figure EV1:**
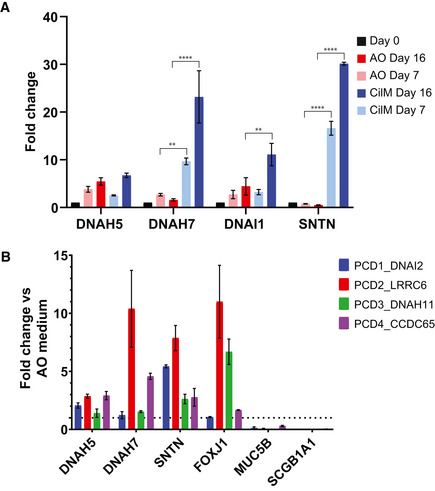
Healthy and PCD AOs in CilM upregulate cilia‐related genes Cilia‐related genes *DNAH5* and *DNAI2* increase over time in AO medium but more strikingly in CilM. Cilia‐related genes *DNAH7* and *SNTN* do not increase over time in AO medium while increasing in CilM. Error bars = stdev. **P < 0.01, ****P < 0.0001 using two‐way ANOVA. *N* = 3.Cilia‐related gene *DNAH5*, *DNAH7*, *SNTN* and *FOXJ1* expression is increased after 14 days in CilM compared to the same donor line in AO medium. The increase shows donor–donor variation. Similarly, the expression of secretory cell markers *MUC5B* and *SCGB1A1* is downregulated in CilM compared to AO medium after 14 days. Error bars = SD. *N* = 3.

**Figure EV2:**
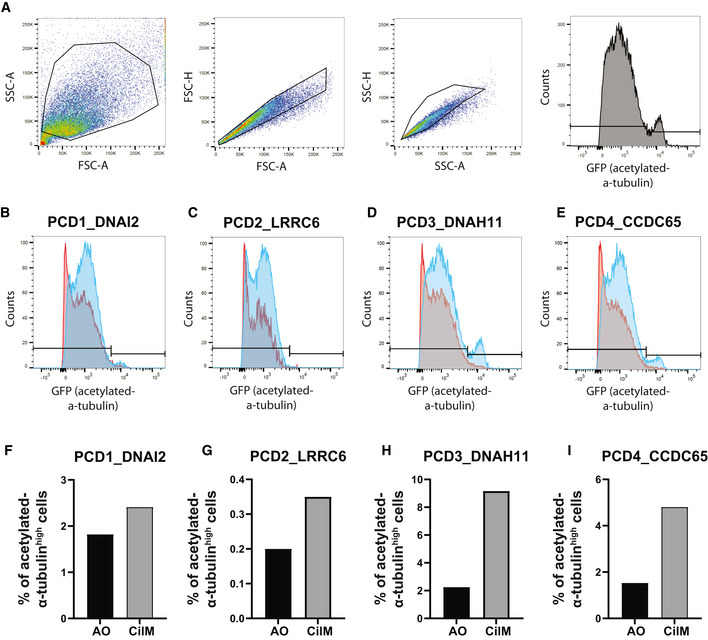
Ciliated cell numbers increase in all PCD AOs with varying efficiency after differentiation in CilM ARepresentative FACS plots show that single acetylated‐α‐tubulin^high^ cells were identified among the population in AOs in CilM and AO medium.B–EThe histograms show differentiation efficiency based on intensity of acetylated‐α‐tubulin expression of the four PCD AO lines. Increased counts of GFP^high^ cells could be identified in the single cell population in AOs differentiated for 14 days in CilM compared to AOs cultured in AO medium. Bar indicates gating strategy for quantification.F–IBar plots depicting percentages of acetylated‐α‐tubulin^high^ cells in AOs differentiated in CilM for 14 days or cultured for 14 days in AO medium. Increased percentages of ciliated cells can be observed in all donor lines with varying fold changes.

To optimize the application of primary AOs in modelling PCD, we aimed to establish a protocol to enhance differentiation towards a ciliated cell fate. Bone morphogenic protein (BMP) and Notch signalling have previously been implied in driving this process (Spassky & Meunier, 2017). We inhibited Notch signalling by adding the gamma‐secretase inhibitor DAPT, removing the BMP signalling inhibitor Noggin and replacing it with recombinant BMP4 in the standard AO culture medium. This media, which we termed “cilia medium” (CilM), induced a strong increase in ciliated cell numbers in the established AOs (Fig 2). The rate of differentiation appeared somewhat variable; full differentiation required 14–21 days. After 21 days in CilM, the apical surface area of AOs that stained for the cilium‐marker acetylated‐α‐tubulin was increased from ± 1% to ± 35% of total surface area (Fig 2A and B). Incubation in CilM arrested the expansion of organoids (Fig 2C). To obtain significant numbers of differentiated cells, we applied the following strategy: first, organoids were allowed to grow into large cysts in AO medium for 1–3 weeks. Next, CilM was added to the cultures for at least 2 weeks. Increasing levels of ciliated cell marker genes were observed over a 16‐day period (Fig EV1A). The cilia could be visualized by live staining of SiR‐tubulin (Movie EV3) and, in fixed organoids, by immunofluorescent staining for acetylated‐α‐tubulin (Fig 2D). SiR‐tubulin‐positive and acetylated‐α‐tubulin‐positive cilia were identified on the luminal side of cystic organoids (Fig 2D), while dense organoids showed little to no cilia. This underlines the importance of cystic organoids in culture. Similar differentiation potential was observed in patient‐derived AOs although some donor–donor variation was observed. While PCD2_LRRC6 AOs showed increased apical presence of acetylated‐α‐tubulin, the percentage was lower than observed in other PCD AOs (Fig 2E). Similarly, this difference in number of ciliated cells was observed using flow cytometry (Fig EV2A). Yet, increased ciliated cell numbers in all PCD AOs was observed after 14 days of CilM compared to AO medium (Fig EV2B–I). Limited numbers are due to the build‐up of the organoids in which only the most apical layer of cells within the organoids can form cilia. Only cells which possess cilia were counted using flow cytometry, while cells in lower cell layers could show ciliated cell characteristics except for cilia formation due to the limited space for cilia build‐up.

**Figure EV3:**
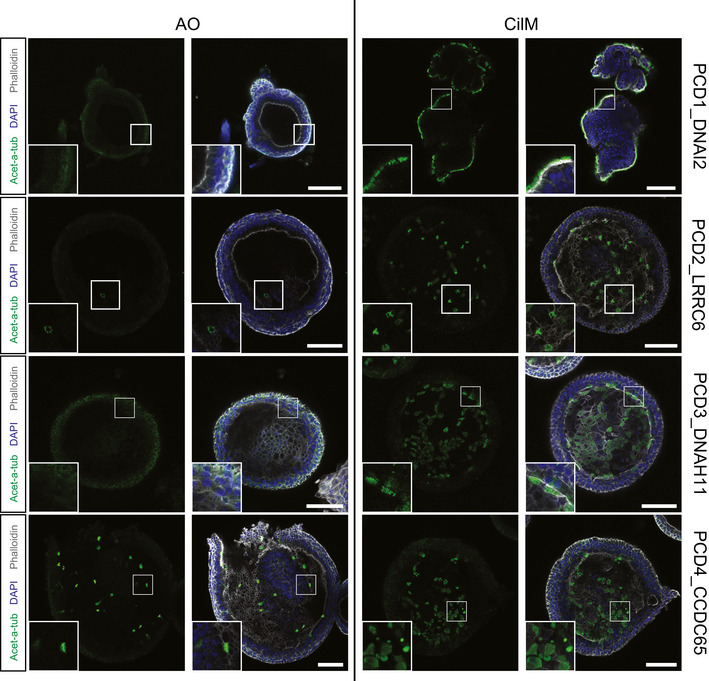
Varying numbers of cilia are visible on the apical surface of PCD AOs An increased number of cilia (acetylated‐α‐tubulin^+^ (acet‐a‐tub)) is observed in patient‐derived PCD AOs after differentiation for 14 days in CilM (right panels) compared to AO Medium (left panels) in all donor lines. Representative images show varying numbers of cilia between donors in both AO medium and CilM. Scale bar = 100 µm. Images of PCD3_DNAH11 are similar to Fig 2F.

Moreover, the presence of acetylated‐α‐tubulin‐positive cells with visible cilia was observed in all donors using immunofluorescence (Figs 2F and EV3). Coordinated beating of the cilia was visualized using the live stain mentioned above (Movie EV3). Taken together, healthy and patient‐derived AOs grown in CilM presented increased ciliated cell numbers, while healthy AOs also displayed regular CBF.

**Figure 3:**
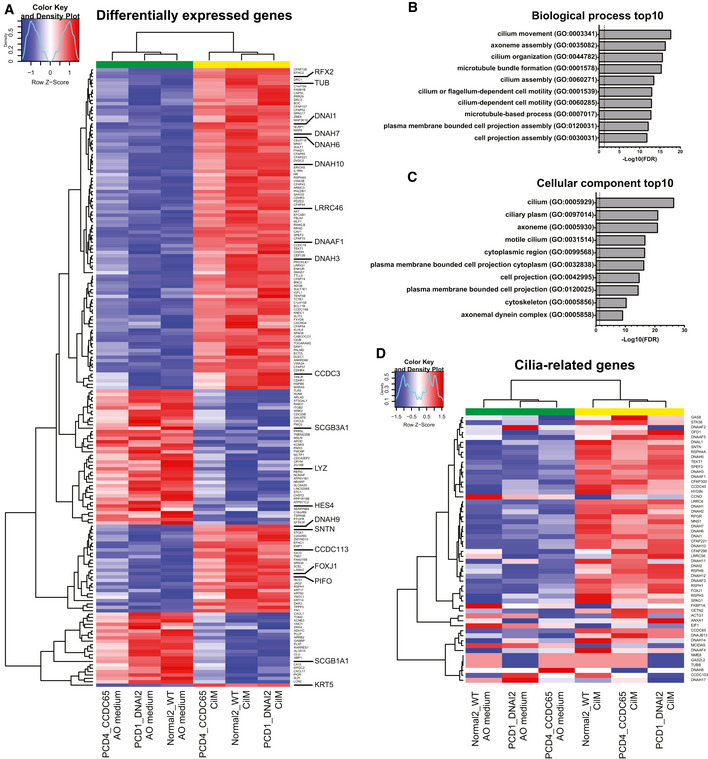
Upregulation of cilia‐related genes in human airway organoids after 21 days in CilM Heatmaps depicting significantly differentially expressed genes of healthy (Normal2_WT) and PCD (PCD1_DNAI2 and PCD4_CCDC65) organoids after 21 days in CilM compared to AO medium. Both healthy and PCD organoids showed an upregulation of ciliated genes such as RFX2, DNAI1 and CCDC3 and a downregulation of marker genes related to secretory cells. *P* < 0.05 and abs(Log2Fold change) > 1.5. Coloured bar represents row *z*‐scores of donor effect corrected normalized counts. CilM AOs (yellow) cluster apart from AO medium AOs (green) in unsupervised hierarchical clustering.GO term enrichment analysis for biological processes of the significantly upregulated genes in CilM compared to AO medium. The top 10 genes are all related to the function of ciliated cells/ cilia function.GO term enrichment analysis for cellular component of the significantly up‐regulated genes in CilM compared to AO medium. The top 10 genes are all related to the function of ciliated cells/cilia function.Heatmaps depicting cilia‐related genes in healthy (Normal2_WT) and PCD (PCD1_DNAI2 and PCD4_CCDC65) organoids after 21 days in CilM compared to AO medium. Both healthy and PCD organoids showed an upregulation of most ciliated genes. Coloured bar represents row z‐scores of donor effect corrected normalized counts. CilM AOs (yellow) cluster apart from AO medium AOs (green) in unsupervised hierarchical clustering.

**Figure EV4:**
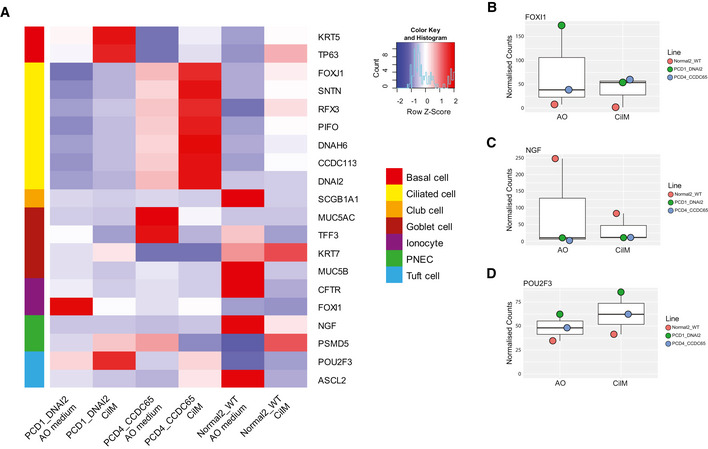
Contribution of PNEC, ionocyte and tuft cells does not change in CilM Heatmaps depicting expression of known cell markers for pulmonary cell types in Normal2_WT, PCD1_DNAI2 and PCD4_CCDC65 cultured in CilM and AO medium. Row colour on the left indicates cell type. PNEC = pulmonary neuroendocrine cell. Coloured bar represents *Z*‐score of log2 transformed values of bulk mRNA sequencing data.No significant differences in normalized counts of ionocyte marker *FOXI1* in Normal2_WT, PCD1_DNAI2 and PCD4_CCDC65 cultured AO or in CilM were observed as indicated in this dotplot graph. Colours of dots indicate the individual organoid line. Boxplot shows median, two hinges (25^th^ and 75^th^ percentile) and two whiskers (largest and smallest value no further than 1.5× inter‐quartile range).No significant differences in normalized counts of pulmonary neuroendocrine cell marker *NGF* in Normal2_WT, PCD1_DNAI2 and PCD4_CCDC65 cultured AO medium or in CilM was observed as indicated in this dotplot graph. Colours of dots indicate the individual organoid line. Boxplot shows median, two hinges (25^th^ and 75^th^ percentile) and two whiskers (largest and smallest value no further than 1.5× inter‐quartile range).A slight increase normalized counts of tuft cell marker *POU2F3* in Normal2_WT, PCD1_DNAI2 and PCD4_CCDC65 cultured in CilM was observed compared to AO medium. Colours of dots indicate the individual organoid line. Boxplot shows median, two hinges (25^th^ and 75^th^ percentile) and two whiskers (largest and smallest value no further than 1.5× inter‐quartile range).

**Figure 4:**
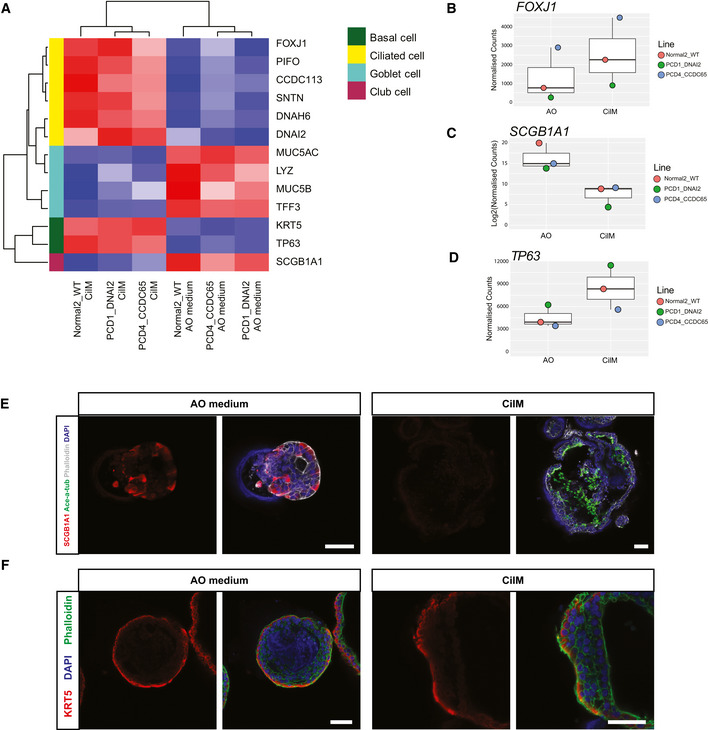
Cellular compositional changes in AOs upon culturing in CilM by bulk mRNA sequencing AHeatmaps depicting the expression of well‐established marker genes for the different pulmonary cell types in normal (Normal2_WT) and PCD (PCD1_DNAI2 and PCD4_CCDC65) organoids, cultured for 21 days in CilM compared to 21 days in AO medium. Coloured bar represents row z‐scores of donor‐corrected normalized counts.B–DDotplot graph depicting the normalized counts of ciliated cell marker *FOXJ1* (B), club cell marker *SCGB1A1* (C) and basal cell marker *TP63* (D) in Normal2_WT, PCD1_DNAI2 and PCD4_CCDC65 cultured for 21 days in CilM or AO medium. Colours of dots indicate the individual organoid line. Boxplot shows median, two hinges (25^th^ and 75^th^ percentile) and two whiskers (largest and smallest value no further than 1.5× inter‐quartile range).ERepresentative immunofluorescence images of PCD AOs (PCD2_LRRC6) in CilM (14 days) stained for cilia (acetylated‐α‐tubulin (Ac‐α‐tub)), secretory cells (SCGB1A1), cellular membrane (Phalloidin) and nucleus (DAPI). Secretory cells can be identified in AOs cultured for 21 days in AO medium as SCGB1A1^+^ cells but not in AOs cultured in CilM for 21 days. In contrast, cilia (acetylated‐α‐tubulin (Ace‐a‐tub)^+^) can only be identified in AOs cultured in CilM. Representative image. Scale bar = 50 μm.FRepresentative image of PCD AOs (PCD3_DNAH11) in CilM (14 days) stained for basal cells (keratin 5 (KRT5)), cellular membrane (Phalloidin) and nucleus (DAPI). No significant difference in the number of basal cells can be observed between organoids cultured in AO medium or CilM (14 days). Scale bar = 50 μm.

**Figure EV5:**
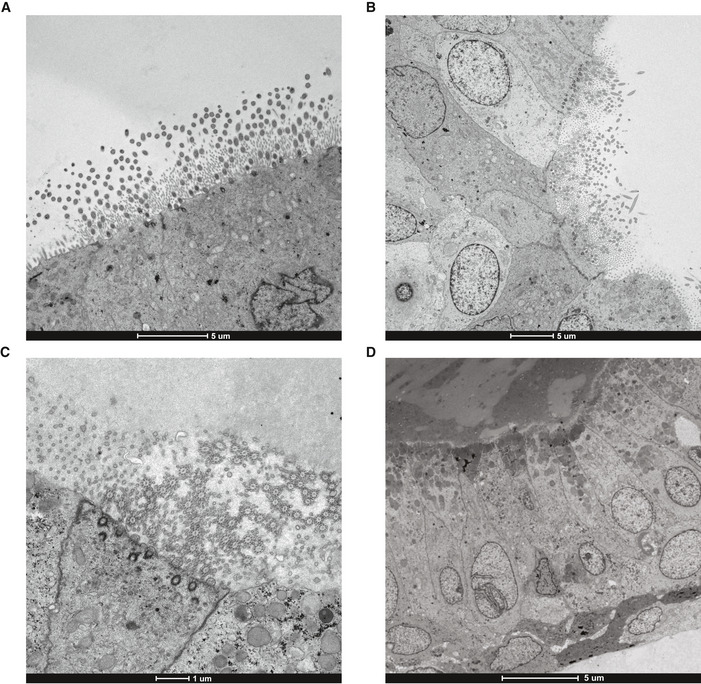
Transmission electron microscopy reveals patches of ciliated cells ACiliated cells were identified facing the lumen of PCD2_LRRC6 AOs in CilM. Scale bar = 5 µm.B, CCiliated cells were more frequently identified in PCD3_DNAH11 AOs in CilM. Scale bar = 5 µm (B) and 1 µm (C).DRare AOs with secretory cells were identified in PCD2_LRRC6 AOs in AO medium. Scale bar = 5 µm.

To better characterize the processes of differentiation towards ciliary fate, bulk mRNA sequencing was performed on three donors (Normal2_WT, PCD1_DNAI2 and PCD4_CCDC65) in CilM and AO medium. Large differences in gene expression were observed in organoids grown in CilM compared to control organoids grown in AO medium (Fig 3A, Appendix Fig S2 and Tables EV1 and EV2). 193 genes were found differentially expressed (130 upregulated and 63 downregulated) (adjusted *P *< 0.01 and abs(log2fold) > 1.5) (Table EV3). Satisfyingly, genes significantly upregulated in CilM organoids were characterized as being involved in “cilium movement” by GO term analysis and as well as in the GO terms “ciliated cell functioning including axoneme assembly” and “cilium assembly” (Fig 3B), as well as “component of the cilium and axoneme” (Fig 3C). We then specifically assessed expression levels of a self‐assembled list of genes that have been described to be involved in cilium structure or intra‐cilium transport (Table EV4) (Horani & Ferkol, 2018). A general increase in the expression of cilia‐related genes was observed in CilM grown organoids from all donors (Fig 3D). Of note, similar to the immunostainings, some donor–donor variation was observed (Appendix Fig S2 and Fig EV4A). PCD4_CCDC65 showed higher levels of ciliated cell marker genes in the generic AO medium compared to the other donors, while these levels still increased in CilM. The data were therefore normalized using batch‐to‐batch variation correction. In general, highly similar transcriptomic changes can be observed when AOs are differentiated in CilM, independent of the AO’s origin (Fig 4A and B, Appendix Fig S2 and Fig EV4A). These findings were verified using RT–qPCR in Normal1_WT AOs (Fig EV1A) and in all four PCD AOs (Fig EV1B) showing the increase of cilia‐related genes *DNAH5*, *DNAH7*, *SNTN*, *DNAI1* and *FOXJ1*. Besides inducing ciliated cell differentiation, CilM lowered expression levels of typical secretory cell markers: *SCGB1A1* (Club cells) and *MUC5B*, *MUC5AC* and *TFF3* (Goblet cells) (Fig 4A and C). Similar downregulation of secretory cell markers *SCGB1A1* and *MUC5B* was observed in the four PCD AO lines after 14 days of differentiation in CilM compared to AO medium (Fig EV1B). Using immunofluorescence, secretory cells could be identified in AO medium cultures (Figs 4E and EV5D). These secretory cells were lost in CilM AOs (Fig 4E). We also noted a general increase of expression of basal cell marker genes (TP63 and KRT5) (Fig 4A and D). No clear differences in KRT5^+^ basal cell number or localization were identified between organoids grown in AO medium or in CilM (Fig 4F). Cell type markers for pulmonary neuroendocrine cells, tuft cells and ionocytes were generally low or undetectable and did not appear to change (Fig EV4B–D). Taken together, CilM induces differentiation of AO cells towards a ciliated cell fate at the cost of secretory cell differentiation.

**Figure 5:**
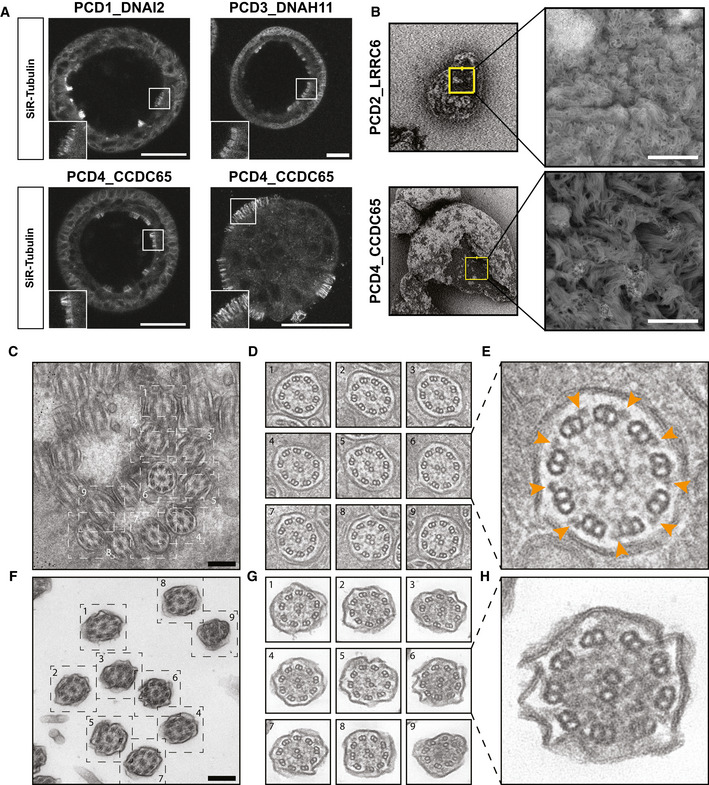
PCD AOs differentiated towards ciliated cells allow live and nanoscopic analysis ARepresentative immunofluorescence images of patient‐derived PCD AOs stained with a SiR‐tubulin live stain. Differentiation in CilM results in an increase in the number of ciliated cells. Scale bar = 50 µm.BScanning electron microscopy images of PCD organoids. Luminal cilia occur in patient‐derived AOs that differentiated in CilM for 21 days. Zoom ins shows cilia. Scale bar = 5 µm.C–HElectron tomography showing the mutant cilia phenotypes from PDC3_DNAH11 (C–E) and PCD2_LRRC6 (F–H) patient‐derived organoids. Dual‐axis tilt series were acquired of which the 0°‐tilt are represented after erasing the fiducial gold beads in IMOD (C, F). After the 3D reconstruction, cilia phenotypes were easily recognized by volume visualization in ChimeraX (D, G). PCD2_LRRC6 AOs showed dynein arm disorders (H), although cilia of PCD3_DNAH11 still had clearly recognizable outer and inner dynein arms (E; arrowheads). Scale bars represent 250 nm.

The presence of ciliated cells in CilM was confirmed for all four PCD AOs using live fluorescent imaging and transmission and scanning electron microscopy (Fig 5). After 21 days in CilM, PCD AOs showed varying, yet abundant levels of ciliated cells in all cystic organoids. The ciliated cells were mostly located on the luminal side of the organoids while some more dense AOs showed an inverse polarity with cilia on the outside of the organoid (Fig 5A). Ciliary immobility was observed in all cystic AOs of PCD1_DNAI2, PCD2_LRRC6 and PCD3_DNAH11 (Movie EV4). Luminal coverage of cilia was confirmed using scanning electron microscopy in patient‐derived AOs (Fig 5B). Transmission electron microscopy identifies patches of ciliated cells in PCD3_DNAH11 and PCD2_LRRC6 organoids differentiated in CilM (Fig EV5A–C). Similar to earlier quantification, PCD2_LRRC6 showed much less cilia when compared to PCD3_DNAH11. Contrarily, rare AOs were identified with patches of cells with secretory vesicles in AO medium PCD2_LRRC6 but never in organoids grown in CilM (Extended data 7d). Closer inspection of the cilia in organoids grown in CilM revealed ultrastructural abnormalities in both PCD3_DNAH11 as in PCD2_LRRC6, as described previously (Horani, Ferkol, *et al*, 2013). PCD3_DNAH11 showed the presence of outer dynein arms but misalignment of the inner arm in the cilia analysed (Fig 5C–E). This contrasted with the outer arm location of DNAH11 protein. Ciliary ultrastructures of the PCD3_DNAH11 patient were indicated as normal during diagnostics. This might indicate the need of additional material in the form of AOs to aid diagnostics. PCD2_LRRC6 missed both inner and outer dynein arms (Fig 5F–H).

Given that for some patients, diagnostic parameters like ciliary ultrastructures remain inconclusive for a definitive PCD diagnosis (Table EV5) and the abundance of ciliated cells in CilM, we then studied cilia function in the PCD patient‐derived AOs. These observations were compared to available diagnostic data of the same patients. All patients had low nasal nitric oxide (nNO) production (12–59 ppb, while the threshold for diagnosis is < 200 ppb). Apart from nNO levels, each patient lacked important additional diagnostic values. Patients 1 (PCD1_DNAI2) and 4 (PCD4_CCDC65) did not present with otitis media, while patient 3 (PCD3_DNAH11) had no signs of bronchiectasis (Table EV5). To include a new diagnostic measure, patient‐specific functional phenotypes were studied in organoids in CilM by 4‐h video recording. While healthy AOs showed directional mucosal spin over this period due to their coordinated ciliary beating, AOs with mutations in the outer dynein arm (PCD1_DNAI2 and PCD3_DNAH11) displayed no ciliary beating, resulting in absent mucosal spin. Surprisingly, both patients presented with inconclusive and normal electron micrograph respectively which could have led to misdiagnosing the patient. Contrarily, PCD3_DNAH11 AOs showed some structural abnormalities in organoid‐derived TEM images which confirmed diagnosis of PCD (Fig 5C–E). Ciliary beating was observed in AOs of PCD4_CCDC65, but mucosal spin remained absent (Movie EV5). The patient presented during diagnosis with a normal electron micrograph. Slow‐motion imaging of the ciliary beat of PCD4_CCDC65 revealed uncoordinated and stiff beating characterized by loss of the arching of the cilium (Movie EV6). This aberrant cilium movement explained the absence of directional mucosal spin in long‐term imaging. Satisfyingly, a PCD phenotype could be identified for all three patients that presented with an inconclusive or a normal electron micrograph. Most importantly, in comparison to ALI cultures, AOs can be expanded over extensive period of time. AO cultures therefore can provide additional and potentially crucial information to the standard PCD diagnosis which involves ALI and electron micrographs and provide additional material for later studies.

To summarize the potential of AOs as diagnostic measures, PCD1_DNAI2, PCD2_LRRC6 and PCD3_DNAH11 showed immotile cilia in the live imaging of AOs while PCD4_CCDC65 showed hyperkinetic and/or stiff ciliary movement. Both in PCD2_LRRC6 as PCD3_DNAH11 AOs, ultrastructural abnormalities were observed which was missed in PCD3_DNAH11 during diagnostics. In all four patients, no mucosal swirl was observed. Together with nNO levels, these parameters were able to identify PCD patient samples apart from healthy (Table EV5). Additional patient‐derived lines will be needed to further strengthen this claim.

**Figure 6:**
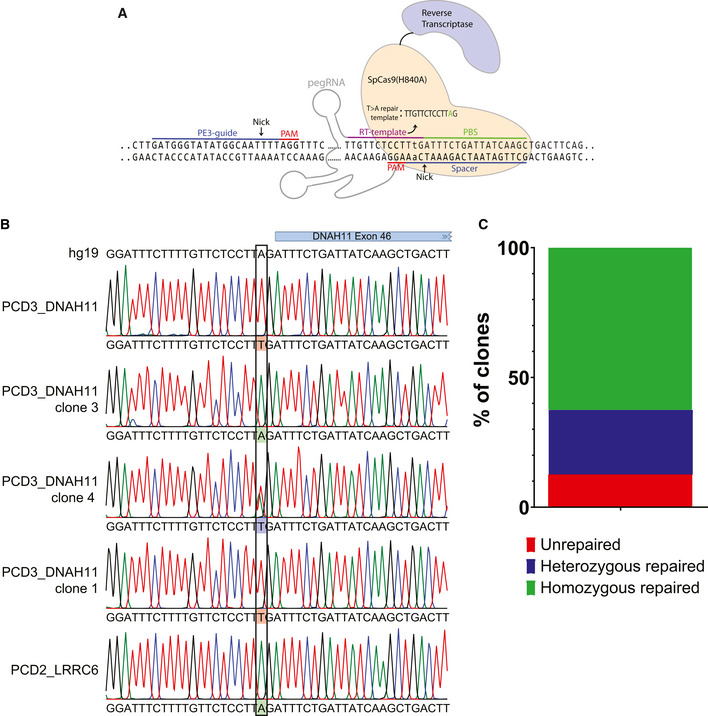
Repair of DNAH11 mutation in PCD3_DNAH11 using prime editing Schematic drawing of the prime editing complex including sequences used for prime editing (PE3)‐guide, reverse transcriptase (RT)‐template, primer binding site (PBS) and spacer. RT‐template indicates the base exchange of T > A to repair c.7472‐2A>T in *DNAH11*.Sanger sequencing traces from isolated single organoid clones aligned to the reference sequence of *DNAH11* (hg19). PCD3_DNAH11 organoids were electroporated with the primer editor. Clones from PCD3_DNAH11 wild type (green), heterozygous (blue) and mutated (red) were identified.Bar plot of quantification of the efficiency of repair with prime editing in picked clones of PCD3_DNAH11. Unrepaired (red), homozygous repaired (green) and heterozygous repaired (blue) sequences could be identified.

Apart from increased diagnostic opportunities, organoids allow for genetic manipulation. Repair of the mutated gene and the future perspective of potential autologous transplantation of the organoids could be regarded as definitive treatment of PCD patients. Therefore, we applied prime editing in PCD AOs (Geurts *et al*, 2020b; Schene *et al*, 2020). PCD3_DNAH11 AOs were transfected with plasmids coding a modified Cas9 with nickase activity and reverse translation of a provided wild‐type DNA template (Fig 6A). To repair the mutation in *DNAH11* in PCD3_DNAH11, organoids were made into single cells, incubated with the above‐mentioned DNA and electroporated using earlier described method (Fujii *et al*, 2019). Outgrowth of clones after recovery and culturing was minimal potentially due to suboptimal conditions. Small AOs that stopped proliferating after several days but survived hygromycin selection, of which resistance was introduced in the same electroporation using the piggyBac system, were genotyped to identify efficiency of the genetic editing. We could identify both homozygous and heterozygous repair of the mutated allele (Fig 6B). 62.5% of the organoids showed homozygous repair, 25% of the organoids showed heterozygous repair, and 12.5% of the AOs picked were resistant to selection but showed no repair (Fig 6C). Unfortunately, none of the repaired organoid lines could be expanded long‐term, something we have observed consistently when AO manipulation involves transfection and a sub‐cloning step. Although optimization on generating stable lines is needed, the AOs allow for genetic editing to repair PCD‐causing mutations.

## Discussion

Here, we describe a long‐term disease modelling strategy using AOs derived from NITB samples. Combined treatment with a Notch inhibitor and a BMP activator (CilM) improves differentiation towards ciliated cells when compared to our original AO protocol (Sachs *et al*, 2019). CilM thereby facilitates easy visualization of ciliated cell‐related biology and disease. Induction of ciliated cells coincides with inhibition of secretory differentiation. This bifurcation is known to be controlled by Notch signalling, a phenomenon exploited in our strategy (Guseh *et al*, 2009). Of note, it is somewhat surprising that Notch inhibition of the intestinal epithelium (both *in vivo* and in organoids) results in the opposite effect: a dramatic increase in secretory lineage cells (Milano *et al*, 2004; Fre *et al*, 2005; Van Es *et al*, 2005; Yin *et al*, 2014). The differentiated AO lines retain their multipotency as evidenced upon re‐transfer to the original AO medium. One advantage of this model system over conventional diagnostic cultures is the possibility for long‐term culture and thereby for more extensive modelling, for example by using CRISPR/Cas9 tools (Driehuis & Clevers, 2017). Currently, AOs show restricted growth after stress by electroporation, lentiviral transduction or subcloning. Expanding these organoids using current conditions do not allow sustained growth. Of note, unmanipulated airway organoids can be expanded > 1–2 years (Sachs *et al*, 2019) and are thus comparable to classical cell lines, while primary cells can only be cultured for weeks, without significant expansion of the input cells.

Research questions that can be answered by this differentiation protocol specifically address ciliated cell function. The AO cultures, however, remain multipotent as evidenced upon re‐transfer to the original AO medium. One advantage of this model system over conventional diagnostic cultures is the possibility for long‐term culture and thereby for more extensive modelling, for example by using CRISPR/Cas9 tools (Driehuis & Clevers, 2017). Increased outgrowth potential is however required to generate stable organoid lines. Currently, AOs show limited growth after stress, like electroporation or lentiviral transduction, and of stop in growth is observed after several days. Expanding these organoids using conventional expansion conditions does not allow sustained growth. The organoids can be expanded > 1 year in expansion conditions (Sachs *et al*, 2019) and are thus comparable to classical cell lines, while primary cells can only be cultured for weeks, without significant expansion of the input cells.

We present an example of ciliated cell disease modelling by using patient‐derived AOs from four PCD patients. Our approach requires a very small amount of donor material as compared to current diagnostic practice and allows for standardized tests for a range of PCD phenotypes. Moreover, we show that the AO cultures can identify ciliary movement defects in cases where electron microscopical analysis would not reveal any ultrastructural ciliary changes. The AO approach can therefore be seen as an additional and potentially definitive diagnostic assay of PCD patients.

To date, treatment options for PCD and other disorders involving motile cilia remain limited. Patient‐derived organoids have proven to predict individual patient responses in cancer (Lee *et al*, 2018; Tiriac *et al*, 2018; Driehuis, Kolders, *et al*, 2019; Driehuis, van Hoeck, *et al*, 2019), as well as in cystic fibrosis (Dekkers *et al*, 2016; de Winter‐De Groot *et al*, 2018; Sachs *et al*, 2019). Treatments for cystic fibrosis can however not be directly translated to PCD (Barbato *et al*, 2009; Lucas & Carroll, 2014; Shapiro *et al*, 2016). PCD‐derived ciliated organoids could be used to identify or develop drugs that target the ciliary defect directly and may facilitate development of gene therapy approaches. Indeed, AOs derived from cystic fibrosis patients have been corrected by CRISPR approaches (Geurts *et al*, 2020a). While transplantation of human stem cell‐derived organoids is not yet feasible, the potential of gene‐correcting PCD patient‐derived AOs followed by autologous transplantation may become a reality. Taken together, PCD AO models may become instrumental to obtain cell‐biological, diagnostic and therapeutic insights into primary ciliary dyskinesia and other ciliated cell‐related diseases.

## Materials and Methods

### Patient samples

Nasal inferior turbinate brushes were obtained from the Hadassah Medical Centre, Jerusalem. All patients were diagnosed with primary ciliary dyskinesia. The study was approved by the ethical committee and was in accordance with the Declaration of Helsinki and according to Israeli law under IRB approval number 075‐16 HMO. This study is compliant with all relevant ethical regulations regarding research involving human participants.

### Organoid establishment

After NITB sample collection, sample was incubated in Advanced DMEM/F12 (Life Technologies; 12634‐034) supplemented with GlutaMax (Life Technologies; 12634‐034), HEPES (Life Technologies; 15630‐056), penicillin (10,000 IU/ml) and streptomycin (10,000 IU/ml) (Life Technologies; 15140‐122) (AdDF+++) with 100 µg/ml Primocin (InvivoGen; ant‐pm1) for transport. Samples were shipped at 4°C overnight to Utrecht, The Netherlands. Human airway cells were isolated, processed and cultured as described previously (Sachs *et al*, 2019). In short, cells were spun down and supernatant was removed. Cells clumps were mechanically sheared using narrowed glass pipette to remove mucus. Cells were resuspended in AdDF+++ supplemented with 100 µg/ml Primocin, 1.25 mM NAc (Sigma‐Aldrich; A9165), 0.15% Pronase E (Sigma‐Aldrich; 7433‐2) and 0.5 mg/ml collagenase (Sigma‐Aldrich, C9407) and incubated for 20 min at 37°C. Samples were washed twice with AdDF+++ with Primocin and spun down at 300× *g* for 5 min. The resulting pellet was resuspended in ice‐cold 70% 10 mg/ml cold Cultrex growth factor reduced BME type 2 (Trevigen; 3533‐010‐02) in airway organoid medium.

### Organoid culture

Organoids were grown in airway organoid medium, previously described (Sachs *et al*, 2019), which consists of AdDF+++ supplemented with 1× B27 supplement (Life Technologies; 17504‐044), 1.25 mM N‐acetyl‐l‐cysteine (Sigma‐Aldrich; A9165), 10 mM nicotinamide (Sigma‐Aldrich; N0636), 500 nM A83‐01 (Tocris; 2939), 5 µM Y‐27632 (Abmole; Y‐27632), 1 µM SB202190 (Sigma‐Aldrich; S7067), 100 ng/ml human FGF10 (PeproTech; 100‐26), 25 ng/ml FGF7 (PeproTech; 100‐19), 1% (vol/vol) RSPO3, and Noggin (produced via the r‐PEX protein expression platform at U‐Protein Express BV). This medium was termed airway medium (AO). For passaging, organoids were collected, washed with DMEM (Life Technologies; 10566016) supplemented with penicillin (10,000 IU/ml) and streptomycin (10,000 IU/ml) (DMEM+P/S) and disrupted either by mechanical shearing or by digestion with TrypLE Express (Life Technologies; 12605‐010). After passaging, organoid fragments were replated in fresh BME. During expansion, medium was replaced twice a week.

### Differentiation of airway organoids towards ciliated cell fate

Ciliated cell differentiated was initiated when organoids had visually formed a lumen (1–3 weeks in AO medium). AO medium was replaced with cilia medium; AO medium from which Noggin and A83‐01 were removed and 10 µM DAPT (Sigma‐Aldrich; D5942) and 10 ng/ml human BMP4 (Peprotech; 120‐05) was supplemented. Organoids were differentiated for 10–21 days by replacing medium 2 times a week.

### Growth of organoids quantification

After dissociation of AOs to single cells using TrypLE Express (Life Technologies; 12605‐010), 20,000 cells were plated in 20 µl droplets of BME and cultured with AO medium for 14 days before starting differentiation. On day 0, 3, 7, 10, 14 and 21 after start of differentiation, cells were collected and counted using cell counter grids (KOVA; 87144E). Average cell number of 3 wells per day was calculated. Results were repeated in three different experiments, and quantifications were performed in a blinded manner.

### Air–liquid interface cultures

Air–liquid interface cultures were established from healthy and patient‐derived airway organoid cultures. AOs were dissociated into single cells using trypsin–EDTA (0.25%; Gibco‐25200).

250,000 cells were seeded on semi‐permeable transwell membranes (Corning‐3378) coated with bovine collagen type I (30 μg/ml; PureCol; Advanced BioMatrix‐#5005). Single cells were seeded in AO growth medium supplemented with 25 ng/ml recombinant human epidermal growth factor (PeproTech; AF‐100‐15) and cultured in submerged conditions. After 4 days, confluent monolayers were cultured in air‐exposed conditions using differentiation medium adapted from Neuberger *et al* (2011) (Neuberger *et al*, 2011). Medium was changed every 4 days.

### RNA isolation, cDNA synthesis and qPCR

Organoids were collected from tissue culture plates and washed twice in 10 ml of DMEM+P/S. RNA was extracted using The Qiagen RNeasy Mini Kit according to protocol. For cDNA synthesis, GoScript Reverse Transcriptase (Promega; A5003) was used according to protocol. qPCRs were performed in 384‐well format using IQ SYBR Green (Bio‐Rad; 1708880). Gene expression was quantified using the ΔΔCt method and normalized by β‐ACTIN using primers listed in Table 1.

**Table 1:** Primers used for RT–qPCR analysis.

Target	Forward	Reverse	Used before in
DNAH5	AGAGGCCATTCGCAAACGTA	CCCGGAAAATGGGCAAACTG	–
DNAH7	ACTTGCAGAATCGCATCCCA	CTCCTCTCCGCTCACTTGTC	Horani, Ferkol, *et al* (2013)
DNAI1	AACGACGGCTGTCCCTAAAG	AGCCTACAAAACGCTCCCTC	(Horani, Ferkol, *et al* (2013)
SNTN	GCTGCAAACCCAATTTAGGA	TGCTCATCAAGTTCAGAAAGGA	Konishi *et al* (2016)
Β‐ACTIN	CATTCCAAATATGAGATGCGTTGT	TGTGGACTTGGGAGAGGACT	–
FOXJ1	CCTGTCGGCCATCTACAAGT	AGACAGGTTGTGGCGGATT	–
SCGB1A1	ACATGAGGGAGGCAGGGGCTC	ACTCAAAGCATGGCAGCGGCA	–
MUC5B	GGGCTTTGACAAGAGAGT	AGGATGGTCGTGTTGATGCG	–

### RNA isolation and RNA sequencing

Total RNA was isolated from organoids that were cultured in AO medium or cilia medium for 21 days after initial growth period in AO medium using the Qiagen RNeasy Mini Kit. The quality and quantity of isolated RNA were checked and measured using the Bioanalyzer 2100 RNA Nano 6000 Kit (Agilent; 5067‐1511). Library preparation was performed with 500 ng of total input RNA using the TruSeq Stranded Total RNA Kit with Ribo‐Zero Human/Mouse/Rat Sets A and B (Illumina; RS‐122‐2201 and RS‐122‐2202). Library quality was checked using the Agilent High‐Sensitivity DNA Kit (5067‐4626) and the Qubit dsDNA HS Assay Kit (Thermo Fisher Scientific; Q32854). Libraries were pooled to a final concentration of 2 nM. Library pools (1.0–1.4 pM) were loaded and sequenced on an Illumina NextSeq system with 2 × 75‐bp high output. After quality control, mapping and counting analyses were performed using our in‐house RNA analysis pipeline v2.1.0 (https://github.com/UMCUGenetics/RNASeq), based on best practices guidelines (https://software.broadinstitute.org/gatk/documentation/article.php?id=3891).

Differential gene expression analysis was performed using the DESeq2 package (Love *et al*, 2014). Batch‐variation correction was applied using batch effect correction of the limma package in DESeq2.

Data are deposited at GEO under GSE158775. Significantly upregulated genes were subjected to functional enrichment analysis for a biological process using the Enrichment analysis tool of Geneontology (http://geneontology.org/). In short, genes names were copied into the analysis tool from the PANTHER Classification System. The 10 biological processes with highest enrichment (after FDR correction and a *P*‐value cut‐off of 0.05) for differentiation are displayed with the corresponding GO term and corrected FDR *P*‐value.

### IHC, fixed whole‐mount IF and live staining

Organoids were collected from tissue culture plates and washed twice in 10 ml of Cell recovery solution (Corning; 734‐0107). Organoids were fixed in 4% paraformaldehyde. For immunohistochemistry, this was followed by dehydration, paraffin embedding, sectioning and standard H&E staining. For whole‐mount immunofluorescence staining, the organoids were permeabilized for 20 min in 0.5% Triton X‐100 (Sigma) and blocked for 45 min in 1% BSA or 2% normal donkey serum. Organoids were incubated with primary antibodies overnight at RT, washed three times with PBS, incubated with secondary antibodies (Invitrogen) and indicated additional stains (DAPI; Life Technologies; D1306 and Phalloidin‐Alexa488; Life Technologies; A12379) 2 h at RT, washed two times with PBS and mounted in VECTASHIELD non‐hard‐set antifade mounting medium (Vector laboratories). For microtubule live stain, organoids were transferred to glass‐bottom plates and incubated with medium containing 100 nM SiR‐tubulin dye (Spirochrome). 12–24 h later, the organoids were imaged.

Samples were imaged on SP8 confocal microscope using LAS X software (all Leica) and processed using ImageJ. Staining was performed on acetylated‐α‐tubulin (Santa Cruz; sc‐23950), actin (Sigma‐Aldrich; A5228), SCGB1A1 (Santa Cruz; sc‐9773) keratin 5 (Covance; PRB 160P‐100).

### Quantification of apical surface coverage

IHC images of acetylated‐α‐tubulin‐stained AOs were loaded into Fiji image software. Using tracing tool, the surface area was calculated. Secondly, the area stained positive was measured. Percentages per organoid were calculated. For PCD AOs differentiation efficiency, each separate experiment shows counts of 10 organoids and quantifications were performed in a blinded manner.

### Flow cytometry

AOs were differentiated using described protocols. On the day of the analysis, organoids were harvested and washed in 10 ml DMEM+P/S. Organoids were dissociated into single cells using TrypLE (Gibco) supplemented with Y‐27632 at 37°C for 30 min. During the single‐cell dissociation, the organoid suspension was vigorously pipetted every 5 min to keep the solution homogenous. Cells were fixed in 4% paraformaldehyde for 2 h at RT. The organoids were permeabilized for 30 min in 0.5% Triton X‐100 (Sigma) and blocked for 30 min in 2% normal goat serum in PBS. Organoids were incubated with primary antibody (mouse‐anti‐acetylated‐α‐tubulin; Santa Cruz; sc‐23950) for 1 h at RT, washed three times with PBS, incubated with secondary antibodies (goat‐anti‐mouse IgG‐Alexa488; Invitrogen) for 1 h at RT and washed two times with PBS. Cells were strained over 35 µm mesh into Falcon® 5 ml Round Bottom Polystyrene Test Tube (Corning; 352235). FACS analysis was performed within 30 min on BD LSR Fortessa X20 4 laser FACS machine. Data analysis was performed using FlowJo (v10.7.2).

### Scanning EM

Organoids were removed from BME, washed with excess AdDF+++, fixed for 15 min with 1% (v/v) glutaraldehyde (Sigma) in phosphate‐buffered saline (PBS) at room temperature and transferred onto 12‐mm poly‐L‐lysine‐coated coverslips (Corning).

Samples were subsequently serially dehydrated by consecutive 10‐min incubations in 2 ml of 10% (v/v), 25% (v/v) and 50% (v/v) ethanol–PBS, 75% (v/v) and 90% (v/v) ethanol–H2O, and 100% ethanol (2×), followed by 50% (v/v) ethanol–hexamethyldisilazane (HMDS) and 100% HMDS (Sigma).

Coverslips were removed from the 100% HMDS and air‐dried overnight at room temperature.

Organoids were manipulated with 0.5‐mm tungsten needles using an Olympus SZX9 light microscope and mounted onto 12‐mm specimen stubs (Agar Scientific).

Following gold coating to 1 nm using a Q150R sputter coater (Quorum Technologies) at 20 mA, samples were examined with a Phenom PRO tabletop scanning electron microscope (Phenom‐World).

### Transmission EM

Organoids were removed from BME, washed with excess AdDF+++ + 5% FBS. Organoids were fixed with 1.5% glutaraldehyde in 0.1 M cacodylate buffer. They were kept in the fixative for 24 h at 4°C. Then, they were washed with 0.1 M cacodylate buffer and postfixed with 1% osmium tetroxide in the same buffer containing 1.5% potassium ferricyanide for 1 h (dark) at 4°C. Then, the samples were dehydrated in ethanol, infiltrated with Epon resin for 2 days, embedded in the same resin and polymerized at 60°C for 48 h. Ultrathin sections of 50 nm were obtained using a Leica Ultracut UCT ultramicrotome (Leica Microsystems, Vienna) and mounted on Formvar‐coated copper grids. They were stained with 2% uranyl acetate in water and lead citrate. Then, sections were observed in a Tecnai T12 electron microscope equipped with an Eagle 4kx4k CCD camera (Thermo Fisher Scientific, the Netherlands).

### Electron tomography

Epon sections were cut with a thickness of 200 nm and 10 nm BSA‐gold fiducial were placed on top to facilitate tomogram alignment. Dual‐axis tomograms were recorded inside a FEI T12 electron microscope running at 120 kV using the FEI automated tomography acquisition software and using 1° tilt increments and a pixel size of 0.94 nm. Tomogram reconstruction was performed with the IMOD software. Projection images of the cilia were extracted using the ChimeraX and 3D volume renders were made with Amira.

### Time‐lapse and high‐speed microscopy

Bright‐field AO time‐lapse videos were recorded at 37°C and 5% CO_2_ on an AF7000 microscope equipped with a DFC420C camera using LAS AF software (all Leica). Bright‐field cilia movement in organoids and air–liquid interface cultures was recorded using the same set‐up equipped with a Hamamatsu C9300‐221 high‐speed CCD camera (Hamamatsu Photonics) at 150 frames per second using Hokawo 2.1 imaging software (Hamamatsu Photonics).

### Plasmid construction for prime editing

Human codon‐optimized prime editing constructs were a kind gift from David Liu: pCMV_PE2 (Addgene plasmid #132775) and pU6‐pegRNA‐GG‐acceptor (Addgene plasmid #132777). The empty sgRNA plasmid backbone was a kind gift from Keith Joung (BPK1520, Addgene plasmid #65777). pegRNA were created as previously described (Anzalone *et al*, 2019). In brief, the pU6‐pegRNA‐GG‐acceptor plasmid was digested overnight using BsaI‐HFv2 (NEB), loaded on a gel and the 2.2 kb band was extracted using the QIAquick Gel extraction kit. Oligonucleotide duplexes for the spacer, scaffold and 3′‐extension with their appropriate overhangs were annealed and cloned into the digested pUF‐pegRNA‐GG‐acceptor by golden gate assembly according to the previously described protocol (Anzalone *et al*, 2019). PE3‐guides were cloned using inverse PCR together using BPK1520 as template and Q5 High fidelity polymerase. Upon PCR clean‐up (Qiaquick PCR purification kit), amplicons were ligated using T4 ligase and Dpn1 (both NEB) to get rid of template DNA. PEgRNA and PE3‐guide combinations were designed using the online web‐tool pegFinder (https://www.nature.com/articles/s41551‐020‐00622‐8). All transformations in this study were performed using OneShot Mach1t1 (Thermo Fisher Scientific) cells and plasmid identity was checked by Sanger sequencing (Macrogen). Spacer and 3′ extensions for PEgRNA and PE3‐Guide can be found in Table 2. Primers for plasmid construction can be found in Table 3.

**Table 2:** Spacer and 3′ extensions for PEgRNA and PE3‐Guide used for Prime editing in AOs.

Plasmid name	Spacer sequence	3′‐ extension
DNAH11‐PEgRNA	GCTTGATAATCAGAAATCaA	TTGTTCTCCTTtGATTTCTGATTATCAAGC
DNAH11‐PE3‐guide	GATGGGTATATGGCAATTTT	–

**Table 3:** Primers used for Prime editing in AOs.

5′ → 3′ sequence	Primer name
CACCGCTTGATAATCAGAAATCaAGTTTT	DNAH11 peg_spacer_F_2
CTCTAAAACTTGATTTCTGATTATCAAGC	DNAH11 peg_spacer_R_2
GTGCTTGTTCTCCTTAGATTTCTGATTATCAAGC	DNAH11 peg_EXTF _2
AAAAGCTTGATAATCAGAAATCTAAGGAGAACAA	DNAH11 peg_EXTR_2
AAAATTGCCATATACCCATCCGGTGTTTCGTCCTTTCCACAAG	DNAH11 pe3_2
/5phos/AGAGCTAGAAATAGCAAGTTAAAATAAGGCT AGTCCGTTATCAACTTGAAAAAGTGGCACCGAGTCG	gRNA_scaffold Top
/5phos/GCACCGACTCGGTGCCACTTTTTCAAGTTGAT AACGGACTAGCCTTATTTTAACTTGCTATTTCTAG	gRNA scaffold bottom

### Organoid electroporation

Organoid electroporation was performed with slight modifications to this previously described protocol (Fujii *et al*, 2015; Geurts *et al*, 2020a). Wild‐type airway organoids derived from PCD patient 3 (PCD3_DNAH11) were maintained in their respective expansion medium up until 24 h before electroporation. 24 h in advance, the expansion medium was switched to electroporation medium which adds rho‐kinase inhibitor Y‐27632 (abmole bioscience) to inhibit anoikis and 1.25% (v/v) DMSO. On the day of electroporation, the organoids were dissociated into single cells using TrypLE (Gibco) supplemented with Y‐27632 at 37°C for 30 min. During the single‐cell dissociation, the organoid suspension was vigorously pipetted every 5 min to keep the solution homogenous. Cells were resuspended in 100 μl of Opti‐MEM™ (Gibco; 11058021) and combined with 10 μl plasmid solution containing 7.5 μg pCMV_PE2_P2A_GFP depending on gene editing strategy and 2.5 μg per guide‐RNA plasmid. Electroporation was performed using NEPA21 with settings described before (Fujii *et al*, 2015). After electroporation, the cells were resuspended in 600 µl BME and plated out in 20 μl droplet/well of a pre‐warmed 48‐well tissue culture plate (Greiner). After polymerization, the droplets were immersed in 250 μl of expansion medium and the organoids were maintained at 37°C and 5% CO2.

### Genotyping of mutations in PCD AOs

PCD3_DNAH11 AOs were grown in expansion condition. DNA was extracted from AOs using Quick‐DNA™ MicroPrep (Zymo research; D3021). PCR on ± 500 bp genomic context was performed using standard Q5^®^ Hot Start High‐Fidelity DNA Polymerase protocol (New England Biolabs; M0493). PCR products were purified using NucleoSpin Gel and PCR Clean‐up kit (Bioke; 740609) and Sanger sequenced by sMacrogen Europe.

### Statistical analysis

Statistical analysis was performed with the GraphPad Prism 8 software.

## Author contributions

JV, LB and HC designed and conceived the study. JV and HC wrote the manuscript. JV, LB and NS cultured the organoid lines and performed organoid related experiments. JV and LB analysed the data. JV, LB, JK and HB embedded organoids and performed immunohistochemistry staining. MHG designed prime editing strategy. WJW and CL‐I performed TEM imaging and KK provided tomographic images. PJP supervised electron microscopy core and helped analyses. KE and AG‐H provided patient tissue samples. HC supervised the project.

## Conflict of interest

H.C. is inventor on several patents related to organoid technology together with N.S.; his full disclosure is given at https://www.uu.nl/staff/JCClevers/.

## Supporting information



Appendix

Expanded View Figures PDF

Table EV1

Table EV2

Table EV3

Table EV4

Table EV5

Movie EV1

Movie EV2

Movie EV3

Movie EV4

Movie EV5

Movie EV6

## Data Availability

Bulk mRNA sequencing data are deposited at GEO under GSE158775 (https://www.ncbi.nlm.nih.gov/geo/) and publicly available.
