# Supplementary material for: Modelling of primary ciliary dyskinesia using patient‐derived airway organoids
Source: EMBO Rep. 2021 Oct 25;22(12):e52058. doi: 10.15252/embr.202052058 (PMC8647008; doi:10.15252/embr.202052058)
Supplement: Supplementary file 2 — Expanded View Figures PDF [file EMBR-22-e52058-s004.pdf]

Expanded View Figures

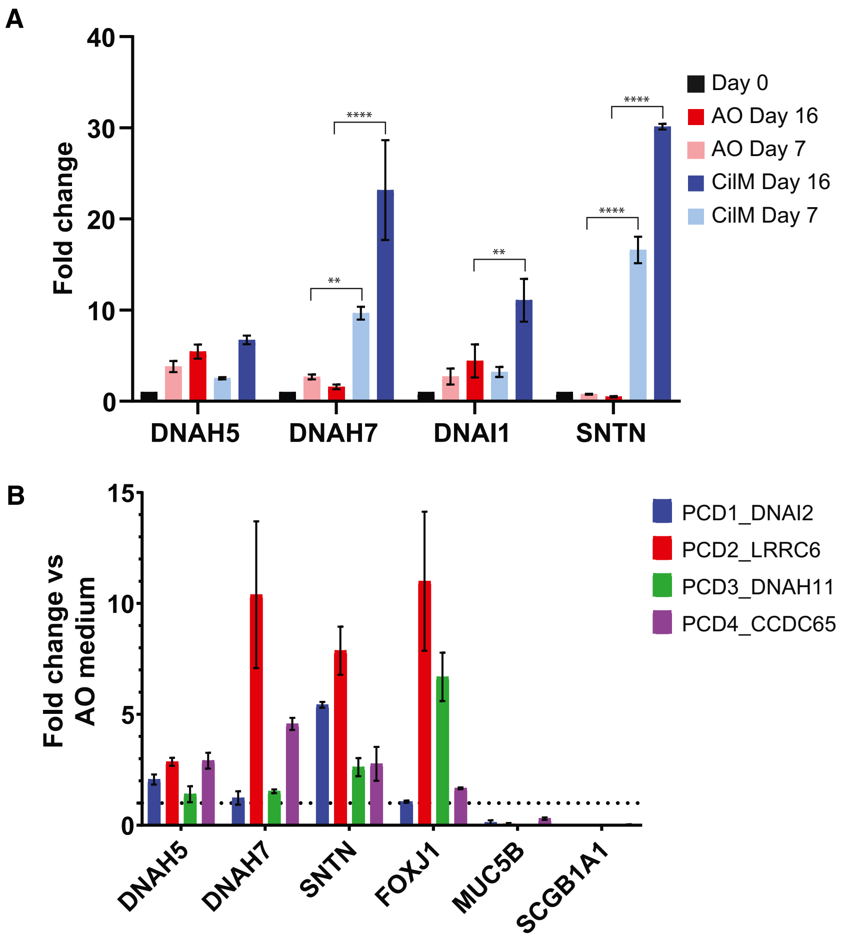

Figure EV1. Healthy and PCD AOs in CilM upregulate cilia-related genes.

A Cilia-related genes *DNAH5* and *DNAI2* increase over time in AO medium but more strikingly in CilM. Cilia-related genes *DNAH7* and *SNTN* do not increase over time in AO medium while increasing in CilM. Error bars = stdev. $^{**}P < 0.01$, $^{****}P < 0.0001$ using two-way ANOVA. $N = 3$.

B Cilia-related gene *DNAH5*, *DNAH7*, *SNTN* and *FOXJ1* expression is increased after 14 days in CilM compared to the same donor line in AO medium. The increase shows donor–donor variation. Similarly, the expression of secretory cell markers *MUC5B* and *SCGB1A1* is downregulated in CilM compared to AO medium after 14 days. Error bars = SD. $N = 3$.

© 2021 The Authors

© 2021 The Authors

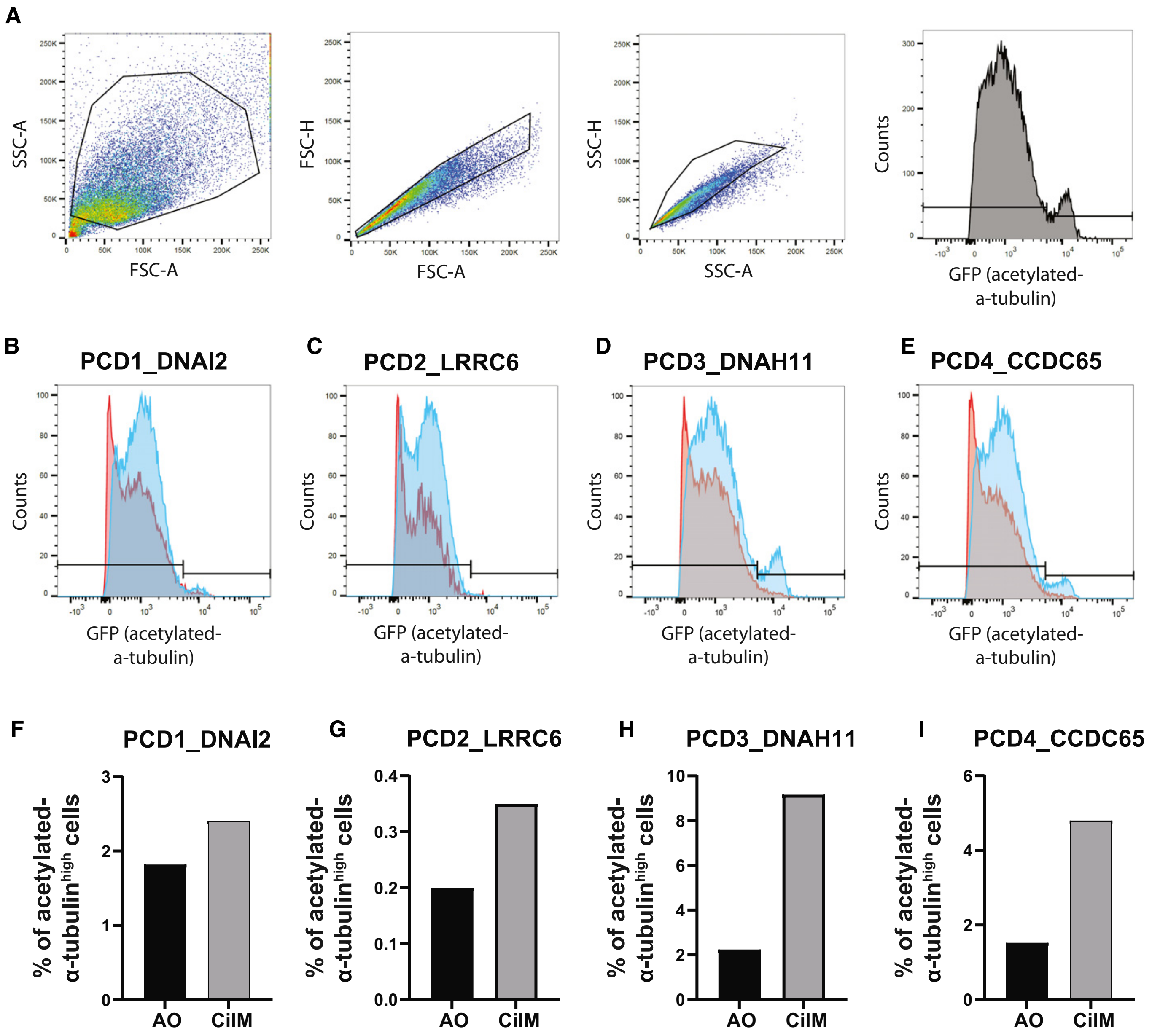

Figure EV2. Ciliated cell numbers increase in all PCD AOs with varying efficiency after differentiation in CilM.

A Representative FACS plots show that single acetylated-α-tubulin^{high} cells were identified among the population in AOs in CilM and AO medium.

B–E The histograms show differentiation efficiency based on intensity of acetylated-α-tubulin expression of the four PCD AO lines. Increased counts of GFP^{high} cells could be identified in the single cell population in AOs differentiated for 14 days in CilM compared to AOs cultured in AO medium. Bar indicates gating strategy for quantification.

F–I Bar plots depicting percentages of acetylated-α-tubulin^{high} cells in AOs differentiated in CilM for 14 days or cultured for 14 days in AO medium. Increased percentages of ciliated cells can be observed in all donor lines with varying fold changes.

 © 2021 The Authors

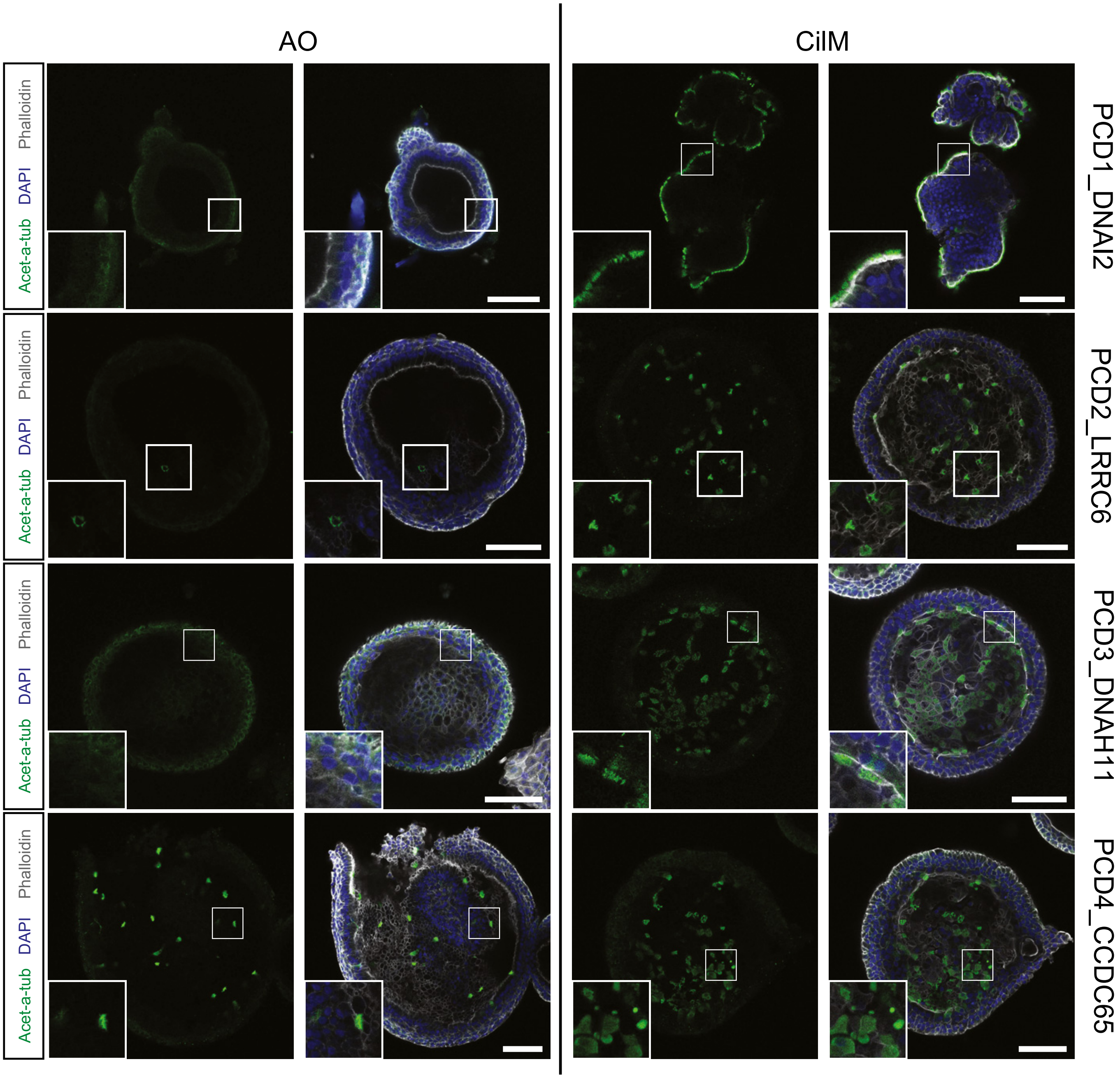

Figure EV3. Varying numbers of cilia are visible on the apical surface of PCD AOs.

An increased number of cilia (acetylated-α-tubulin^{+} (acet-a-tub)) is observed in patient-derived PCD AOs after differentiation for 14 days in CilM (right panels) compared to AO Medium (left panels) in all donor lines. Representative images show varying numbers of cilia between donors in both AO medium and CilM. Scale bar = 100 μm. Images of PCD3_DNAH11 are similar to Fig 2F.

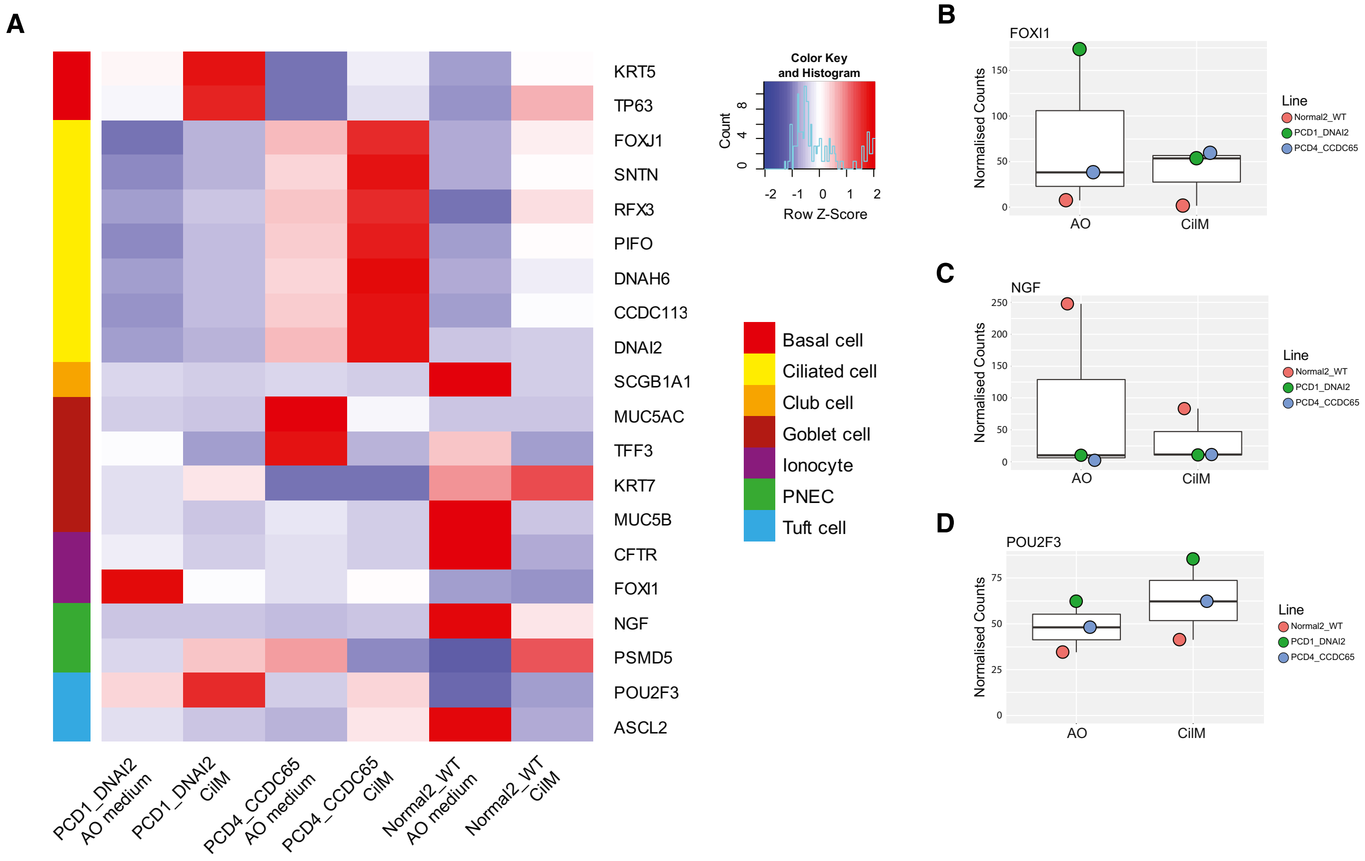

Figure EV4. Contribution of PNEC, ionocyte and tuft cells does not change in CilM.

A Heatmaps depicting expression of known cell markers for pulmonary cell types in Normal2_WT, PCD1_DNAI2 and PCD4_CCDC65 cultured in CilM and AO medium. Row colour on the left indicates cell type. PNEC = pulmonary neuroendocrine cell. Coloured bar represents *Z*-score of log2 transformed values of bulk mRNA sequencing data.

B No significant differences in normalized counts of ionocyte marker *FOXI1* in Normal2_WT, PCD1_DNAI2 and PCD4_CCDC65 cultured AO or in CilM were observed as indicated in this dotplot graph. Colours of dots indicate the individual organoid line. Boxplot shows median, two hinges (25th and 75th percentile) and two whiskers (largest and smallest value no further than 1.5× inter-quartile range).

C No significant differences in normalized counts of pulmonary neuroendocrine cell marker *NGF* in Normal2_WT, PCD1_DNAI2 and PCD4_CCDC65 cultured AO medium or in CilM was observed as indicated in this dotplot graph. Colours of dots indicate the individual organoid line. Boxplot shows median, two hinges (25th and 75th percentile) and two whiskers (largest and smallest value no further than 1.5× inter-quartile range).

D A slight increase normalized counts of tuft cell marker *POU2F3* in Normal2_WT, PCD1_DNAI2 and PCD4_CCDC65 cultured in CilM was observed compared to AO medium. Colours of dots indicate the individual organoid line. Boxplot shows median, two hinges (25th and 75th percentile) and two whiskers (largest and smallest value no further than 1.5× inter-quartile range).

 © 2021 The Authors

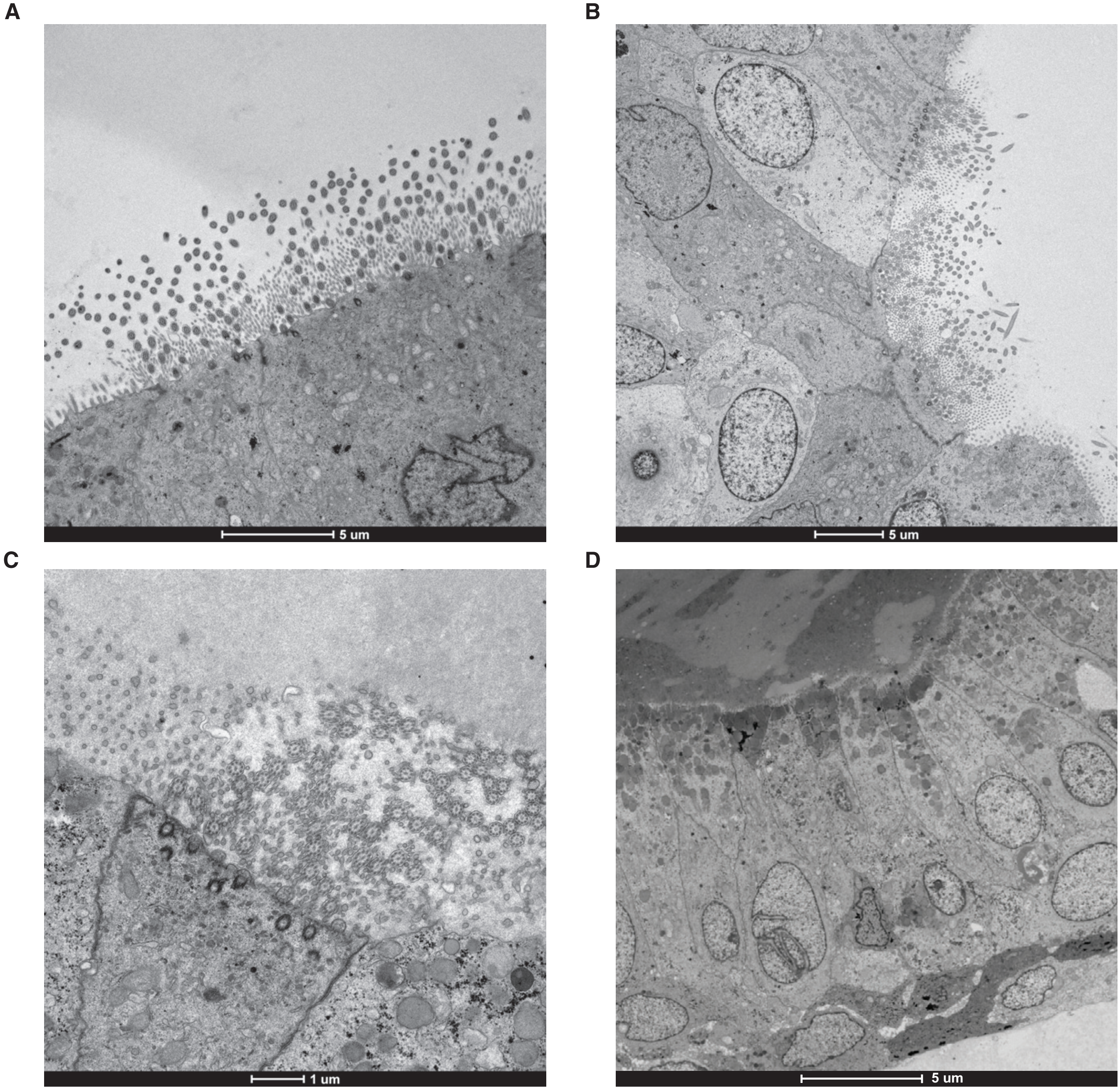

Figure EV5. Transmission electron microscopy reveals patches of ciliated cells.

A Ciliated cells were identified facing the lumen of PCD2_LRRC6 AOs in CilM. Scale bar = 5 μm.

B, C Ciliated cells were more frequently identified in PCD3_DNAH11 AOs in CilM. Scale bar = 5 μm (B) and 1 μm (C).

D Rare AOs with secretory cells were identified in PCD2_LRRC6 AOs in AO medium. Scale bar = 5 μm.

© 2021 The Authors
