# Supplementary material for: Modelling of primary ciliary dyskinesia using patient‐derived airway organoids
Source: EMBO Rep. 2021 Oct 25;22(12):e52058. doi: 10.15252/embr.202052058 (PMC8647008; doi:10.15252/embr.202052058)
Supplement: Supplementary file 8 — Movie EV1 [file EMBR-22-e52058-s010.zip › EMBOR-2020-52058V3-Movie_EV1/Movie EV1.docx]

**Movie EV1. Ciliated cells of primary cilia dyskinesia derived airway organoid show immobility.**

A) Healthy AOs (Normal2_WT) show normal CBF as indicated by SiR-tubulin live stain.

B) PCD patient-derived AOs (PCD3_DNAH11) show ciliary immotility by SiR-tubulin live stain.
