# Supplementary material for: Modelling of primary ciliary dyskinesia using patient‐derived airway organoids
Source: EMBO Rep. 2021 Oct 25;22(12):e52058. doi: 10.15252/embr.202052058 (PMC8647008; doi:10.15252/embr.202052058)
Supplement: Supplementary file 9 — Movie EV2 [file EMBR-22-e52058-s014.zip › EMBOR-2020-52058V3-Movie_EV2/Movie EV2.docx]

**Movie EV2. Air-liquid-interface cultures recapitulate ciliary immotility of PCD patient-derived airway organoids.**

A-B) Slow motion imaging of ALI cultures of healthy AOs (Normal2_WT) indicates normal ciliary beating.

C-D) Slow motion imaging of ALI cultures of PCD AOs (PCD2_LRRC6) indicate ciliary immotility.
