# Supplementary material for: Modelling of primary ciliary dyskinesia using patient‐derived airway organoids
Source: EMBO Rep. 2021 Oct 25;22(12):e52058. doi: 10.15252/embr.202052058 (PMC8647008; doi:10.15252/embr.202052058)
Supplement: Supplementary file 10 — Movie EV3 [file EMBR-22-e52058-s003.zip › EMBOR-2020-52058V3-Movie_EV3/Movie EV3.docx]

**Movie EV3. Airway organoids in CilM show coordinated ciliary beating of differentiated ciliated cells**

Ciliary beating visualised by SiR-Tubulin staining of healthy AOs (Normal1_WT) cultured in CilM indicate large surface coverage of ciliated cells.
