# Supplementary material for: Modelling of primary ciliary dyskinesia using patient‐derived airway organoids
Source: EMBO Rep. 2021 Oct 25;22(12):e52058. doi: 10.15252/embr.202052058 (PMC8647008; doi:10.15252/embr.202052058)
Supplement: Supplementary file 11 — Movie EV4 [file EMBR-22-e52058-s005.zip › EMBOR-2020-52058V3-Movie_EV4/Movie EV4.docx]

**Movie EV4. PCD Airway organoids in CilM show ciliary immobility of differentiated ciliated cells**

Ciliary immobility visualised by SiR-Tubulin staining of PCD AOs (PCD2_LRRC6) cultured in CilM indicate PCD phenotype of ciliated cells.
