# Supplementary material for: Modelling of primary ciliary dyskinesia using patient‐derived airway organoids
Source: EMBO Rep. 2021 Oct 25;22(12):e52058. doi: 10.15252/embr.202052058 (PMC8647008; doi:10.15252/embr.202052058)
Supplement: Supplementary file 12 — Movie EV5 [file EMBR-22-e52058-s002.zip › EMBOR-2020-52058V3-Movie_EV5/MovieEV5.docx]

**Movie EV5. Airway organoids in CilM show coordinated ciliary beating of differentiated ciliated cells and reveal patient-specific phenotypes**

A-D) Long-term brightfield imaging of 4 hours of AOs in CilM shows mucosal spin in healthy AOs (Normal1_WT) (A) and absence in PCD AOs PCD1_DNAI2 (B), PCD3_DNAH11 (C) and PCD4_CCDC65 (D). Ciliary beating can however be identified in Normal1_WT (A) and PCD4_CCDC65 (D).
