# Supplementary material for: Modelling of primary ciliary dyskinesia using patient‐derived airway organoids
Source: EMBO Rep. 2021 Oct 25;22(12):e52058. doi: 10.15252/embr.202052058 (PMC8647008; doi:10.15252/embr.202052058)
Supplement: Supplementary file 13 — Movie EV6 [file EMBR-22-e52058-s006.zip › EMBOR-2020-52058V3-Movie_EV6/Movie EV6.docx]

**Movie EV6. Patient-specific phenotype revealed in AOs in CilM**

Slow motion imaging of ciliary beating of PCD AOs (PCD4_CCDC65) reveals stiff and uncoordinated patterns.
