# Supplementary material for: Modelling of primary ciliary dyskinesia using patient‐derived airway organoids
Source: EMBO Rep. 2021 Oct 25;22(12):e52058. doi: 10.15252/embr.202052058 (PMC8647008; doi:10.15252/embr.202052058)
Supplement: Supplementary file 1 — Appendix [file EMBR-22-e52058-s013.pdf]

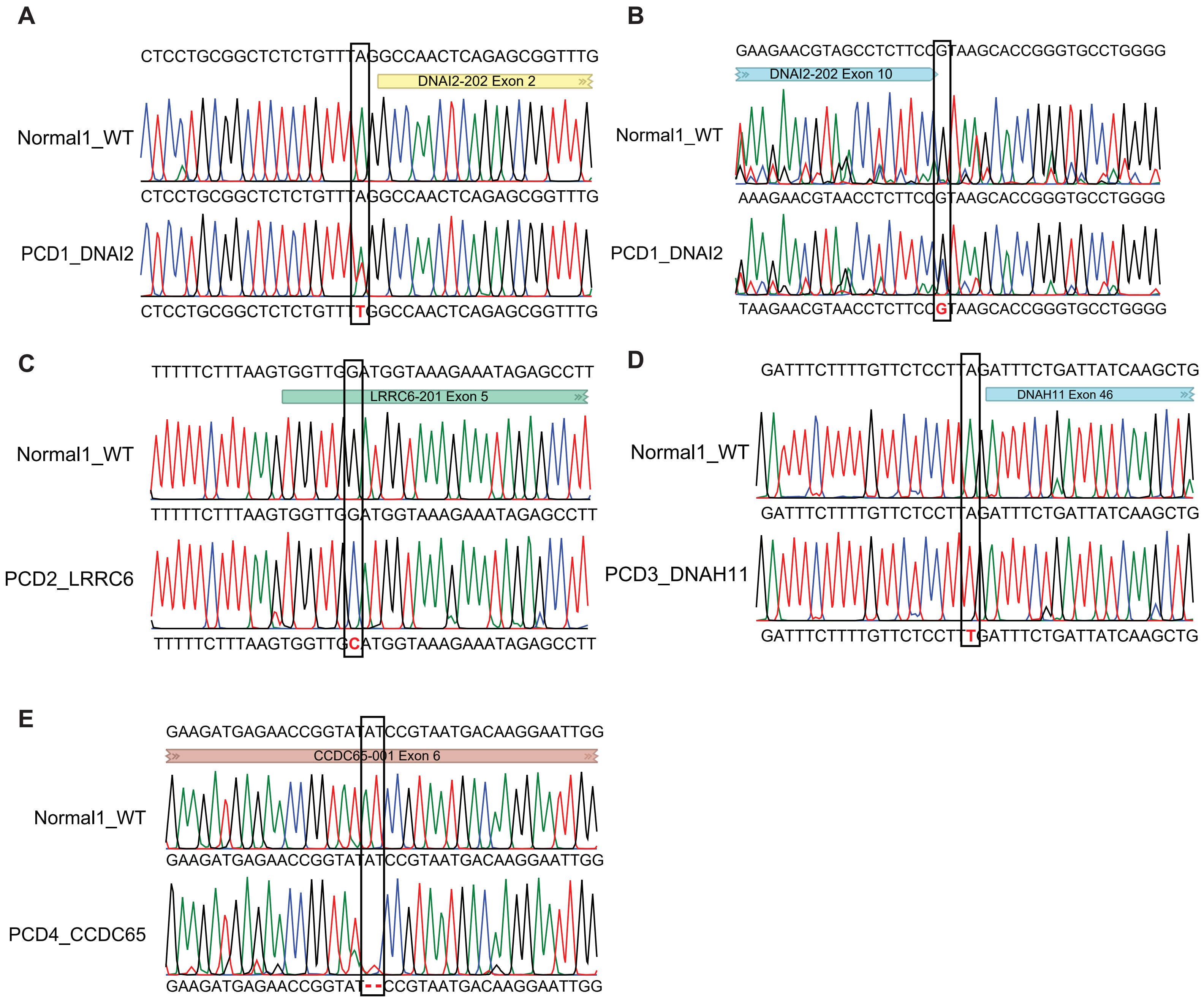

Appendix Figure S1. Mutations in various genes identified in PCD AOs

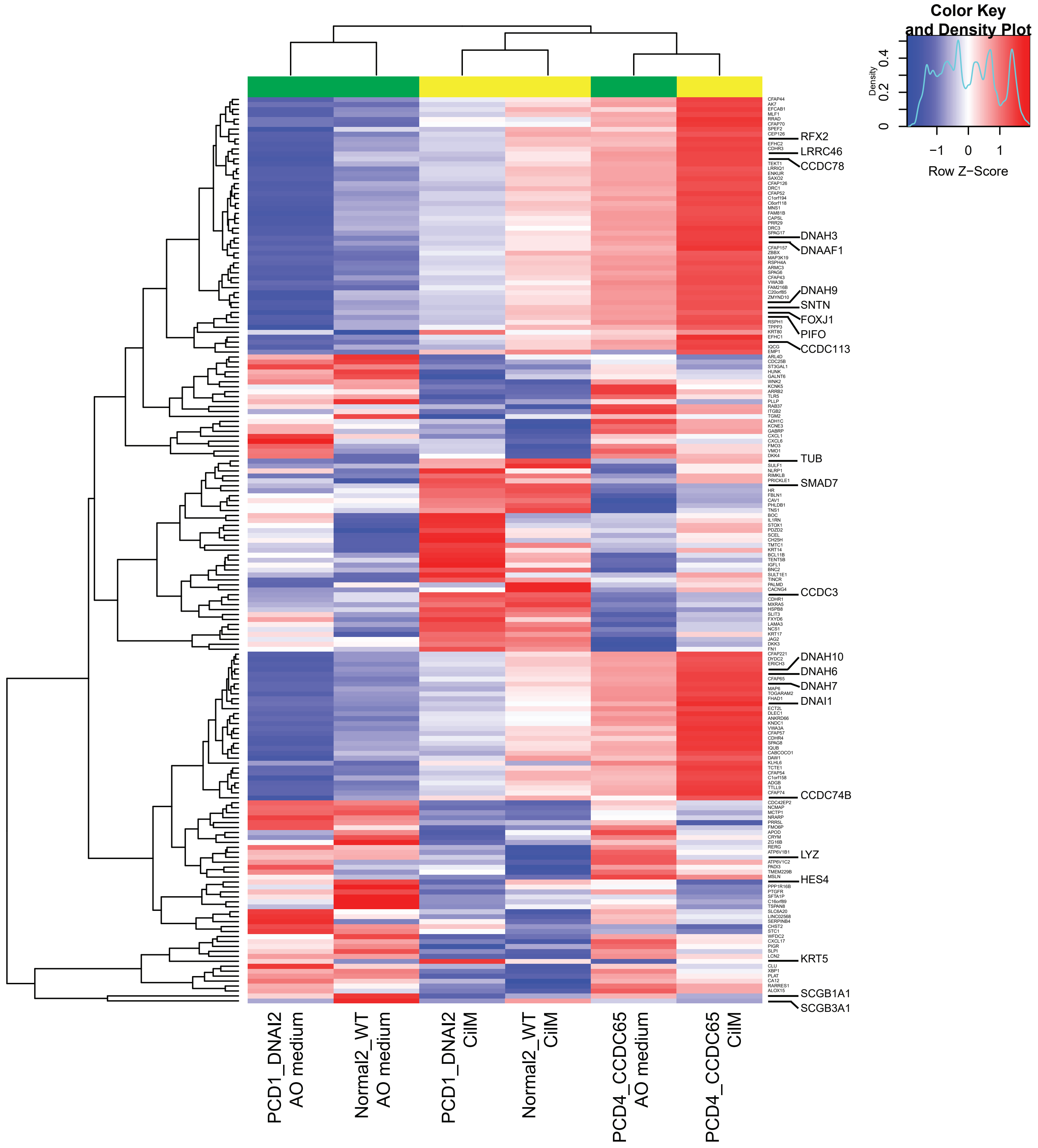

Appendix Figure S2. Differentially expressed genes show donor-donor variation in expression levels but show similar trends in all donors in CilM compared to AO medium
