# Supplementary material for: Modelling of primary ciliary dyskinesia using patient‐derived airway organoids
Source: EMBO Rep. 2021 Oct 25;22(12):e52058. doi: 10.15252/embr.202052058 (PMC8647008; doi:10.15252/embr.202052058)
Supplement: Supplementary file 14 [file EMBR-22-e52058-s001.pdf]

EMBO
reports

Modelling of Primary Ciliary Dyskinesia using Patient-derived Airway Organoids

Jelte van der Vaart, Lena Böttinger, Maarten H. Geurts, Williine J. van de Wetering, Kèvin Knoops, Norman Sachs, Harry Begthel, Jeroen Korving, Carmen Lopez-Iglesias, Peter J. Peters, Kerem Eitan, Alex Gileles-Hillel, Hans Clevers

Review timeline:

Submission date:	09.11.20
Editorial Decision:	09.12.20
Revision received:	06.08.21
Editorial Decision:	16.09.21
Revision received:	19.09.21
Accepted:	21.09.21

Editor: Esther Schnapp

Transaction Report:

(Note: With the exception of the correction of typographical or spelling errors that could be a source of ambiguity, letters and reports are not edited. The original formatting of letters and referee reports may not be reflected in this compilation.)

1st Editorial Decision 9th December 2020

Dear Hans,

Thank you for the transfer of your manuscript to EMBO reports. We have now received the enclosed comments by the referees.

As you will see, all referees acknowledge that the study is potentially interesting. However, they also all point out that significant revisions will be required before the study can be considered for publication here. Together, they raise a number of points and I wanted to share all with you now. If you like, we can discuss the exact revision requirements further by email or video chat. I think that the points raised by referee 1 should be addressed, as well as all textual changes and discussions. I also agree that the AO should be characterized more thoroughly.

I would thus like to invite you to revise your manuscript with the understanding that the referee concerns must be fully addressed and their suggestions taken on board. Please address all referee concerns in a complete point-by-point response. Acceptance of the manuscript will depend on a positive outcome of a second round of review. It is EMBO reports policy to allow a single round of major revision only and acceptance or rejection of the manuscript will therefore depend on the completeness of your responses included in the next, final version of the manuscript.

Revised manuscripts should be submitted within three months of a request for revision; they will otherwise be treated as new submissions. Please contact us if a 3-months time frame is not sufficient for the revisions so that we can discuss this further. You can either publish the study as a short report or as a full article. For short reports, the revised manuscript should not exceed 27,000 characters (including spaces but excluding materials & methods and references) and 5 main plus 5 expanded view figures. The results and discussion sections must further be combined, which will help to shorten the manuscript text by eliminating some redundancy that is inevitable when discussing the same experiments twice. For a normal article there are no length limitations, but it should have more than 5 main figures and the results and discussion sections must be separate.

Regarding data quantification, please specify the number "n" for how many independent experiments were performed, the bars and error bars (e.g. SEM, SD) and the test used to calculate p-values in the respective figure legends. This information must be provided in the figure legends. Please also include scale bars in all microscopy images.

IMPORTANT NOTE: we perform an initial quality control of all revised manuscripts before re-review. Your manuscript will FAIL this control and the handling will be DELAYED if the following APPLIES:
1) A data availability section providing access to data deposited in public databases is missing. If you have not deposited any data, please add a sentence to the data availability section that explains that.

2) Your manuscript contains statistics and error bars based on n=2. Please use scatter blots in these cases. No statistics should be calculated if n=2.

When submitting your revised manuscript, please carefully review the instructions that follow below. Failure to include requested items will delay the evaluation of your revision.

1) a .docx formatted version of the manuscript text (including legends for main figures, EV figures and tables). Please make sure that the changes are highlighted to be clearly visible.

2) individual production quality figure files as .eps, .tif, .jpg (one file per figure). See https://wol-prod-cdn.literatumonline.com/pb-assets/embo-site/EMBOPress_Figure_Guidelines_061115-1561436025777.pdf for more info on how to prepare your figures.

3) We replaced Supplementary Information with Expanded View (EV) Figures and Tables that are collapsible/expandable online. A maximum of 5 EV Figures can be typeset. EV Figures should be cited as 'Figure EV1, Figure EV2" etc... in the text and their respective legends should be included in the main text after the legends of regular figures.

- For the figures that you do NOT wish to display as Expanded View figures, they should be bundled together with their legends in a single PDF file called *Appendix*, which should start with a short Table of Content. Appendix figures should be referred to in the main text as: "Appendix Figure S1, Appendix Figure S2" etc. See detailed instructions regarding expanded view here: <https://www.embopress.org/page/journal/14693178/authorguide#expandedview>

- Additional Tables/Datasets should be labeled and referred to as Table EV1, Dataset EV1, etc. Legends have to be provided in a separate tab in case of .xls files. Alternatively, the legend can be supplied as a separate text file (README) and zipped together with the Table/Dataset file.

4) a .docx formatted letter INCLUDING the reviewers' reports and your detailed point-by-point responses to their comments. As part of the EMBO Press transparent editorial process, the point-by-point response is part of the Review Process File (RPF), which will be published alongside your paper.

5) a complete author checklist, which you can download from our author guidelines <https://www.embopress.org/page/journal/14693178/authorguide>. Please insert information in the checklist that is also reflected in the manuscript. The completed author checklist will also be part of the RPF.

6) Please note that all corresponding authors are required to supply an ORCID ID for their name upon submission of a revised manuscript (<https://orcid.org/>). Please find instructions on how to link your ORCID ID to your account in our manuscript tracking system in our Author guidelines <https://www.embopress.org/page/journal/14693178/authorguide#authorshipguidelines>

7) Before submitting your revision, primary datasets produced in this study need to be deposited in an appropriate public database (see https://www.embopress.org/page/journal/14693178/authorguide#datadeposition). Please remember to provide a reviewer password if the datasets are not yet public. The accession numbers and database should be listed in a formal "Data Availability" section placed after Materials & Method (see also https://www.embopress.org/page/journal/14693178/authorguide#datadeposition). Please note that the Data Availability Section is restricted to new primary data that are part of this study. * Note - All links should resolve to a page where the data can be accessed. *
If your study has not produced novel datasets, please mention this fact in the Data Availability Section.

8) We would also encourage you to include the source data for figure panels that show essential data. Numerical data should be provided as individual .xls or .csv files (including a tab describing the data). For blots or microscopy, uncropped images should be submitted (using a zip archive if multiple images need to be supplied for one panel). Additional information on source data and instruction on how to label the files are available at <https://www.embopress.org/page/journal/14693178/authorguide#sourcedata>.

9) Our journal also encourages inclusion of *data citations in the reference list* to directly cite datasets that were re-used and obtained from public databases. Data citations in the article text are distinct from normal bibliographical citations and should directly link to the database records from which the data can be accessed. In the main text, data citations are formatted as follows: "Data ref: Smith et al, 2001" or "Data ref: NCBI Sequence Read Archive PRJNA342805, 2017". In the Reference list, data citations must be labeled with "[DATASET]". A data reference must provide the database name, accession number/identifiers and a resolvable link to the landing page from which the data can be accessed at the end of the reference. Further instructions are available at https://www.embopress.org/page/journal/14693178/authorguide#referencesformat

We would also welcome the submission of cover suggestions, or motifs to be used by our Graphics Illustrator in designing a cover.

As part of the EMBO publication's Transparent Editorial Process, EMBO reports publishes online a Review Process File (RPF) to accompany accepted manuscripts. This File will be published in conjunction with your paper and will include the referee reports, your point-by-point response and all pertinent correspondence relating to the manuscript.

You are able to opt out of this by letting the editorial office know (emboreports@embo.org). If you do opt out, the Review Process File link will point to the following statement: "No Review Process File is available with this article, as the authors have chosen not to make the review process public in this case."

I look forward to seeing a revised version of your manuscript when it is ready.

© European Molecular Biology Organization

Please let me know if you have questions or comments regarding the revision.

Best regards,
Esther

Esther Schnapp, PhD
Senior Editor
EMBO reports

Referee #1:

Jelte van der Vaart et al. utilized the human airway organoid (AOs) models to study ciliary phenotypes in primary ciliary dyskinesia (PCD) patient derived samples. Specifically, the authors developed a protocol to induce the differentiation of AOs towards a ciliated cell fate by modulating Notch (DAPT), and BMP4 in their cultures (cilia medium' (CilM). Further, they performed differential gene expression analysis by bulk mRNA sequencing to characterize the processes of differentiation towards ciliary fate. Additionally, the authors employed scanning electron microscopy and live imaging to study structural organization and movement of cilia. Overall, the data presented in this manuscript attempt to model cellular defects in primary ciliary dyskinesia using the airway organoid model. However, the enthusiasm is hampered by the lack of novelty, for example, PCD mutations have been well characterized using 2D airway epithelial culture models. As outlined below in my comments, the authors can combine their organoid technology with the CRISPR-Cas or Prime editing tools to correct the patient specific mutation and study whether that can reverse the cilia phenotype, which can potentially strengthen the current manuscript.

1. In the current manuscript authors generated airway organoids from 4 PCD patient samples (each with a specific mutation). As mentioned in their introduction "human airway organoid (AO) culture has opened the possibility of studying airway diseases in patient-specific primary material in models that can be manipulated and studied". Essentially, authors can utilize the PCD patient specific mutant organoids to correct the mutation (at least one immotile mutant) by employing genome editing tools. Additionally, further characterize if the mutation corrected organoid would generate normal cilia and function normally, and whether they are phenotypically comparable to healthy controls.

2. Figure 2, the authors induced healthy human AOs differentiation into ciliated cells using Cilia Medium and quantified the % acetylated α-tubulin+ cells as a measure of differentiation. It is important to do similar measurements in PCD mutant organoids. Also, how does the differentiation look compare to the healthy controls? Similar to extended Data Fig. 1A how is the gene expression pattern for Cilia-related genes in PCD mutant organoids? A quantitative comparison would help in understanding the differences.

3. Figure 1A. Authors used the terminologies such as many, little, few, and some cystic organoids. It is confusing as to what does this represents. A more,

quantitative measure should be included to define these terminologies. Similarly, for the regular, fast, and slow growth.

Referee #2:

Van der Vaart et al have extended their previous studies developing airway organoids, applying the technique to PCD patient cells. Primary nasal brushings from PCD patients sent from Israel were developed as submerged culture organoids with cilia primarily projecting into the spherical organoid lumen as a non-air exposed culture system. They have developed culture conditions that favour ciliated cells and characterised the cultures using cell immunofluorescence, high speed video and transcriptomics. This has revealed a mixed dataset showing variation across samples.

The paper is very focussed on the authors prior work (in organoids, in gut) rather than work in modelling PCD in airways. The introduction background explains about PCD, is very short on developing any text around the development of these organoids, this should be addressed in the introduction as well with a review of the general approach and best methods out there. More discussion comparing the organoid system and existing PCD models would improve this paper.

The PCD mutation nomenclature is insufficient, as presented in the main table. It should follow the usual HGVS guidelines (DNA and protein level change, transcript indicated, add the literature/dbSNP rs number or ClinVar reference, for variants previously published) in order to enhance the evidence these are all PCD-causing. A ClinVar entry can be found for the CCDC65 and the LRRC6 variants which are both previously published but the DNAI2, DNAH11 appear to affect splice sites and seem not previously reported - they need to be clearly indicated, using the usual +1 and -2 nomenclature that seem to apply.

Figure 1a does not show genome sequencing results, evidence for the genetic results need to be included.

It is stated 'for some patients, diagnostic parameters were inconclusive for a definitive 141 PCD diagnosis', yet this is not properly explained - please provide the details of what is meant. No TEM data is shown, for either the original patient diagnostics or the comparative organoid cilia defects. A supplementary table lists the patient features, at least the dynein arm defect of DNAI2 and LRRC6 models would be needed here. In particular, DNAH11 is an apparently novel splice effect variant with immotile cilia - cilia are expected to be hyperkinetic motion for DNAH11 mutations. This seems to still need further evidence as a causing mutation, such as segregation data or splicing defect proof.

On a similar line, the movies should show all of the different patient mutation cilia beating images, since these are four different mutation types causing different PCD cilia beating defects. Currently only one example from the four different patient types is shown in videos 1 and 2, while it is stated that all have been imaged using high speed video. As DNAH11 mutations do not immobilise the cilia but show a hyperkinetic beating pattern, yet immotile cilia were observed - was there no

motility at all in this culture?

The text at the end of results is quite confusing, firstly the mention of nNO, ototits media, bronchiectasis, these things do not seem relevant as not testable in organoids. A simple summary of this text, put into a table, would be more useful. The supplementary table S4 could be moved into main text and the organoid comparison just added into this table, to make clear what is beign said here - it is not clear yet, what advantage the organoid has offered, over ALI cultures? Please clarify this final para.

The majority of the organoids were cystic, what is the relevance of this variability or is this just observation and a variation of the technique? Organoids are a debated model, apart from being a submerged model, as they suppress the mucus secreting cell lineages in favour of ciliated cells, there is not a great deal of comment about this yet from all the genetic profiling that has been done here, about validity of the model compared to transcriptomics that has been done in recent years for the airways, using single cells methods - how does it compare? The authors can expand a little, on their thoughts around how physiological the organoids are as a lung epithelial model, especially as a submerged not an air-exposed system.

In Fig.3, a simple question arises, as to why the PCD organoids are not showing any downregulation of the respective mutated genes in PCD4 and PCD6 lines (CCDC65 and LRRC6). Also, these control versus patient cells are showing up similar red/blue up/down regulated genes - yes, but it is not possible to distinguish the PCD samples from the control - as the two PCD look so dis-similar to each other. These transcriptomic results look hard to decipher for their true value - the huge number of differently expressed genes, 17,000+ genes in S1, is not discussed in any depth (does not give confidence of a meaningful cut-off, unless they have a lot more data generated for more samples and controls than 1). GO annotation has been done for all genes, but then this should be added to the supplementary gene tables S1 and S2 then. In these supplementary tables, column titles are also not separated correctly for Table S1, S2.

What can we conclude about differences between these samples from this data? Top 10 GO term enrichment analysis, this is not explained. Table S2 is an extracted list of ciliary genes. This has not been properly explained.

Fig 4, again it would be reassuring to know that PCD patient cells are viewed as any different from control or not, the 3 lines shown seem quite varied. It is stated that genes upregulated in CilM organoids were characterised as being involved in 'cilium movement' by GO-term analysis and as well as in the GO terms 'ciliated cell functioning including axoneme assembly' and 'cilium assembly' - is this statement fully true, for all three lines displayed, why is data selected for two patients only?

What happened to donors PCD1 and PCD2? If other cultures failed this should be documented.

The characterisation in Fig 5 is quite weak. The only functional evidence of preserved phenotype in PCD is that the mucous doesn't move as it does in normal organoids. No ciliary beat pattern or frequency is given, where is the data? The text

has mentioned nNO, but this has not been measured in the organoids. Also no cilia structural analysis is performed on the organoids?

Minor

1. Instead of just cell line PCD3,4,5,6 it would be more help if these names could incorporate the relevant PCD gene as well, to make more clear for reading in the text, what is expected in terms of motility and cilia structural defect.

2. Intro mentions respiratory distress due to narrowing of the airway, but bronchiectasis typically is characterised by widening and scarring of the airway.

3. 'waxing and waning' is not a clear description, this needs an edit.

4. typo 'relates', 3rd line of Results.

5. Ensure correct variant annotation throughout. This needs to mention transcript e.g. CCDC65 NM_033124.4:c.877_878delAT (any gene symbols in italics).

Referee #3:

Summary
This research paper by van der Vaart et al entitled "Modelling of Primary Ciliary Dyskinesia using Patient-derived Airway Organoids" demonstrates the utility of airway organoids (AO) derived from nasal brushings as a cell-based model that can be used to study PCD and potentially find new therapies. Here, the authors described a method to generate AO from cells isolated from nasal epithelia. These AO are expandable when cultured in "expansion media" (AO media) and further differentiate into ciliated cell lineages in media containing BMP4 and DAPT (CilM). This is demonstrated through SiR-Tubulin live imaging of "beating" cilia and staining for acetylated alpha-tubulin. Importantly, AOs can be generated from nasal brushings from individuals harboring mutations affecting motile cilia function or assembly (PCD3-6). Live imaging of cilia from PCD AO shows disrupted function. Finally, the authors perform bulk sequencing of the AO in AO media versus CilM to show that CilM supports differentiation of the cell to ciliate cells at the expense of secretory cell lineages.
Tissue-specific organoids have become an increasingly important model to study development and pathological causes of disease. PCD, a pulmonary disease caused by dysfunctional motile cilia leading to impaired mucociliary transport of foreign objects has been linked to genetic mutations, of which there are now >40 disease causing mutations. Methods to diagnose PCD are limited and I agree with the authors that there is a need to develop cell-based models for diagnosis and therapy discovery. While this paper does contain some convincing data to support the utility of the AO to model PCD as mentioned above, my enthusiasm for this work is dampened by several major factors: The novelty of the work is lacking, additional validation of the model is needed, and the claim that AO can be used for PCD diagnostics is a big stretch, at least with the current methods shown here. There is not sufficient data presented to support that the model is truly reflective of the airway phenotype let alone it can be used to model PCD airway disease. For this

paper to be accepted, significant additions are needed with careful detailed analyses of the model.

Major weaknesses:

1) Novelty of the work - Developing AO from nasal brushings is not new, neither is the differentiation of the AO to ciliated cells using the sample factors described in the protocol. Perhaps the novelty here is the demonstration that AO can be used to model PCD but this leads to the second major point.

2) The need for additional validation of the model. While the authors provide some characterization of their AO model with bulk sequencing and staining for cilia, additional validation especially of the mutant phenotypes should be done. The data as is, is not sufficient to convince me that the model is appropriate and can truly be used in understanding PCD pathogenesis/diagnosis.
I. Specific staining for dynein proteins is needed to demonstrate genetic-phenotype effects of the mutations and how the phenotype impacts the axoneme.
II. Demonstrate axoneme impairment with electron micrographs and functional analyses of the impaired motile cilia.
III. Snapshot images of SiR-Tubulin stained cilia to demonstrate "immotile cilia" is not convincing. Can the authors use other assays to measure motility? Ie. Mapping fluorescent bead movement?
IV. Can editing the mutation in these AO restore motile cilia function? This might be an indirect method to validate their model.

3) The rationale for differentiation of the AO is unclear. Manipulating the Notch and BMP pathways have previously been shown to induce ciliated cell differentiation and the authors use these approaches to "increase" ciliated cell differentiation in the AO. It is unclear to me what is the rationale for doing so? Is it to increase the number of ciliated cells to monitor function (ie beating)? As a future goal to model patient-specific responses or for therapy discovery, does manipulating the cell type in the AO truly reflective of PCD disease?
I. The bulk sequencing data in Figure 3 appears to be missing half of the data. Specifically, both figure legend and in the results section of the paper, the authors described a difference in gene expression of AO in AO media versus CilM media for each cell line tested and yet it is unclear what the heatmap is showing other than 3 columns representing the 3 cell lines with what appears to be similar expression patterns of genes for AO or CilM? One cannot evaluate the data if the data is not there.
II. Immunostaining, at the very least, is needed to validate the lineages suggested by gene expression. It is hard to know whether a uniform homogenous AO population is formed or there are heterogenous AOs where differences in cell compositions and if so, how does this affect modelling of PCD?
III. While using nasal brushings may be good surrogates of the airway epithelium, the authors should present data to support this. It is unclear if nasal epithelia-derived organoids can model airway epithelial responses.
IV. Clarify the novelty of the differentiation protocol and how it relates to previous publications using similar media components to generate ciliated cells.

Minor weaknesses:

1) This essentially is an n=1 study for each cell line from 1 nasal brushing sample for each mutation and control. Cell composition and cell number will change with each nasal brushing. Are these results reproducible with additional brushings. Similarly, are these results reproducible with additional nasal brushings from other individuals with the same mutation?
2) Authors claim that the AOs can be expanded for >1 year (stated in the discussion). Where is the data to support this claim? And does the phenotype (cell composition, function) of the AOs change with serial passaging/freeze-thaw? Does the karyotype change over time? I am not convinced that primary cells have the capacity for such long-term serial passaging and culture without entering senescence or acquiring oncogenic mutations. The authors should carefully characterise the "long-term" AOs and present this data.
3) Figures 2A, 2D and 2E are showing the same thing...cilia in the AO. This is not an efficient use of space as it seems redundant. Instead the authors should consider providing additional characterization of the ciliated AO. Ie. Data to show that AO after >1 year maintains the phenotype of early AO.
4) Quantification of the number of ciliated cells and other cell types by FACS is a better measure than acetylated tubulin positively over surface area (Figure 2B).
5) Showing presence of cilia in Figure 5 in AO from PCD patients does not indicate "PCD phenotypes". Additional data is needed here to support this.
6) The authors make a statement in the discussion "...we describe a long-term disease modelling strategy using AOs...". How does the present data support this statement?
7) The statement "The AO approach can therefore be seen as an additional and potentially definitive diagnostic assay of PCD patients, abolishing the need, in some cases, for multiple inconclusive diagnostic tests." is a bit of a stretch. This needs to be revised.

© European Molecular Biology Organization

1st Authors' Response to Reviewers 6th August 2021

Referee #1:

Jelte van der Vaart et al. utilized the human airway organoid (AOs) models to study ciliary phenotypes in primary ciliary dyskinesia (PCD) patient derived samples. Specifically, the authors developed a protocol to induce the differentiation of AOs towards a ciliated cell fate by modulating Notch (DAPT), and BMP4 in their cultures (cilia medium' (CilM). Further, they performed differential gene expression analysis by bulk mRNA sequencing to characterize the processes of differentiation towards ciliary fate. Additionally, the authors employed scanning electron microscopy and live imaging to study structural organization and movement of cilia. Overall, the data presented in this manuscript attempt to model cellular defects in primary ciliary dyskinesia using the airway organoid model. However, the enthusiasm is hampered by the lack of novelty, for example, PCD mutations have been well characterized using 2D airway epithelial culture models. As outlined below in my comments, the authors can combine their organoid technology with the CRISPR-Cas or Prime editing tools to correct the patient specific mutation and study whether that can reverse the cilia phenotype, which can potentially strengthen the current manuscript.

1. In the current manuscript authors generated airway organoids from 4 PCD patient samples (each with a specific mutation). As mentioned in their introduction "human airway organoid (AO) culture has opened the possibility of studying airway diseases in patient-specific primary material in models that can be manipulated and studied". Essentially, authors can utilize the PCD patient specific mutant organoids to correct the mutation (at least one immotile mutant) by employing genome editing tools. Additionally, further characterize if the mutation corrected organoid would generate normal cilia and function normally, and whether they are phenotypically comparable to healthy controls.
A: We agree that genome editing of our mutant organoids strengthens our study. Recently, a new technique named prime editing was developed by Liu and colleagues and employed in organoids (Anzalone et al., 2019; Geurts et al., 2020; Schene et al., 2020)*. We have performed prime editing on multiple lines and obtained corrected organoids for PCD3_DNAH11 (hsLung109-PCD5 in previous manuscript). These data are included in figure 6 and lines 236-251. Unfortunately, despite significant efforts over several years, we have not yet been able to turn such corrected lines after the necessary single cell cloning step into stable organoid lines (which we can routinely do in lines derived from gut or liver). This current limitation is discussed in the discussion in line 272-275.*

2. Figure 2, the authors induced healthy human AOs differentiation into ciliated cells using Cilia Medium and quantified the % acetylated α-tubulin+ cells as a measure of differentiation. It is important to do similar measurements in PCD mutant organoids. Also, how does the differentiation look compare to the healthy controls? Similar to extended Data Fig. 1A how is the gene expression pattern for Cilia-related genes in PCD mutant organoids? A quantitative comparison would help in understanding the differences.

A: We agree that differentiation of patient-derived AOs might differ from the healthy controls. We have therefore performed similar analyses on the patient lines and included this in figure 2E which is discussed in line 141-143. Apart from the quantification using IHC, we have included immunostaining images of all lines in figure 2F and Figure EV3 and flow cytometry measurements in Figure EV2. These images and quantifications do show variability between donors but clearly demonstrate increases of ciliated cell numbers when compared to organoids from the same donor in expansion (AO) medium.

3. Figure 1A. Authors used the terminologies such as many, little, few, and some cystic organoids. It is confusing as to what does this represents. A more, quantitative measure should be included to define these terminologies. Similarly, for the regular, fast, and slow growth.
A: Well taken. Since this data does not represent differentiation status or growth rate, which is important for later data representation, we've removed the column from the figure and briefly state the observations in the text in line 114-115 and 140.

Referee #2:

Van der Vaart et al have extended their previous studies developing airway organoids, applying the technique to PCD patient cells. Primary nasal brushings from PCD patients sent from Israel were developed as submerged culture organoids with cilia primarily projecting into the spherical organoid lumen as a non-air exposed culture system. They have developed culture conditions that favour ciliated cells and characterised the cultures using cell immunofluorescence, high speed video and transcriptomics. This has revealed a mixed dataset showing variation across samples.

The paper is very focussed on the authors prior work (in organoids, in gut) rather than work in modelling PCD in airways. The introduction background explains about PCD, is very short on developing any text around the development of these organoids, this should be addressed in the introduction as well with a review of the general approach and best methods out there. More discussion comparing the organoid system and existing PCD models would improve this paper.
A: Well taken. An additional paragraph is added describing the current model systems employed for modelling PCD. This is described in line 67-76. We believe that the additional value of a patient-specific human model as made possible by organoid technology is thus illustrated.

The PCD mutation nomenclature is insufficient, as presented in the main table. It should follow the usual HGVS guidelines (DNA and protein level change, transcript indicated, add the literature/dbSNP rs number or ClinVar reference, for variants previously published) in order to enhance the evidence these are all PCD-causing. A ClinVar entry can be found for the CCDC65 and the LRRC6 variants which are both previously published but the DNAI2, DNAH11 appear to affect splice sites and seem not previously reported - they need to be clearly indicated, using the usual +1 and -2 nomenclature that seem to apply.

A: Well taken. Additional information about the exact location of the found mutation has been included in figure 1a as well as a reference to known dbSNP numbers.

Figure 1a does not show genome sequencing results, evidence for the genetic results need to be included.
A: We have now included Sanger sequencing traces of the mutations in Appendix Figure S1.

It is stated 'for some patients, diagnostic parameters were inconclusive for a definitive 141 PCD diagnosis', yet this is not properly explained - please provide the details of what is meant.
A: We have added a reference to Table EV5 in which these characteristics are listed in line 206, 205-211. We have included threshold data to clarify which parameters are in line with PCD diagnosis and which are inconclusive or represent normal values. We have compared these diagnostic parameters with the data gathered in this study and discussed this in line 229-234.

No TEM data is shown, for either the original patient diagnostics or the comparative organoid cilia defects. A supplementary table lists the patient features, at least the dynein arm defect of DNAI2 and LRRC6 models would be needed here.
A: Well taken. We have included TEM images of PCD2_LRRC6 (hsLung108-PCD4 in previous manuscript) and PCD3_DNAH11 (hsLung109-PCD5) AOs in CilM which indicate the abnormalities expected and published in PCD2_LRRC6 (Horani et al., 2013) as well as in PCD3_DNAH11 AOs (figure 5C-H). These findings are discussed in line 192-201.

In particular, DNAH11 is an apparently novel splice effect variant with immotile cilia - cilia are expected to be hyperkinetic motion for DNAH11 mutations. This seems to still need further evidence as a causing mutation, such as segregation data or splicing defect proof.
A: Ciliary immobility of DNAH11-mutated PCD3_DNAH11 is shown in figure 1D and Video EV1B. While we agree that novel mutations found in DNAH11 will be interesting to follow up, the scope of the manuscript is to generate a broad model system which allows for representative follow-up studies like the one proposed. We therefore believe it is outside the scope of this manuscript to phenotype this mutation in DNAH11 in greater detail.

On a similar line, the movies should show all of the different patient mutation cilia beating images, since these are four different mutation types causing different PCD cilia beating defects. Currently only one example from the four different patient types is shown in videos 1 and 2, while it is stated that all have been imaged using high speed video. As DNAH11 mutations do not immobilise the cilia but show a hyperkinetic beating pattern, yet immotile cilia were observed - was there no motility at all in this culture?
A: While some reports indeed show the hyperkinetic phenotype of DNAH11 mutations, the mutation found in the patients from which PCD3_DNAH11 was generated is novel, as mentioned by the reviewer. Effects may differ between individual mutations, underlining the need for patient-specific model systems. As seen in figure 1d and Video EV1, the cilia are fully immotile. Indeed, we have never

observed any motility in PCD3_DNAH11 cilia. This could be explained by the lack or misalignment of inner dynein arms seen in TEM data (Figure 5c-e).

The text at the end of results is quite confusing, firstly the mention of nNO, otitits media, bronchiectasis, these things do not seem relevant as not testable in organoids. A simple summary of this text, put into a table, would be more useful.
A: Agreed. The summary is given in Table EV5 and is now referenced in the text in line 206 and 205-211.

The supplementary table S4 could be moved into main text and the organoid comparison just added into this table, to make clear what is beign said here - it is not clear yet, what advantage the organoid has offered, over ALI cultures? Please clarify this final para.
A: We agree that the comparison between ALI cultures and AOs has not been fully discussed. We have added some extra remarks to clarify the differences in lines 74-77 and 224-227. Most significantly, the AOs can be maintained and expanded over long time periods, while the ALI cultures are static and relatively short-lived (Hirst et al., 2010; Hirst et al., 2014). *Moreover, mucosal spin can only be identified in a 'closed' 3D AO. ALI cultures would have not immediately identified the patient-specific default in PCD4_CCDC65 (hsLung111-PCD6 in previous manuscript) since it would have required extensive imaging of the cilia. The 3D AOs can identify PCD phenotypes without the need for additional techniques like slow-motion imaging and can be generated from small biopsies.*

The majority of the organoids were cystic, what is the relevance of this variability or is this just observation and a variation of the technique?
A: We mention this specifically because ciliated cells appear in all cystic organoids (which contain all major differentiated cell types), but are only rarely present in dense organoids (which consist primarily of progenitor cells). Moreover, the mucosal swirl can only be identified in the cystic organoids. Therefore, a high ratio of cystic organoids is important for easy visualisation.
This is now more explicitly stated in the text in line 138-140.

Organoids are a debated model, apart from being a submerged model, as they suppress the mucus secreting cell lineages in favour of ciliated cells, there is not a great deal of comment about this yet from all the genetic profiling that has been done here, about validity of the model compared to transcriptomics that has been done in recent years for the airways, using single cells methods - how does it compare? The authors can expand a little, on their thoughts around how physiological the organoids are as a lung epithelial model, especially as a submerged not an air-exposed system.
A: The airway organoids used in this manuscript have been extensively characterised (Sachs et al., 2019) *and tend to generate secretory cells more than ciliated cells. The current study adapts our original culture protocol to improve the generation of ciliated cells.*
Of course, the luminal side of postnatal lung epithelium is exposed to air, a situation not represented in our culture system. However, continuous expansion of these (and all other organoid types that our lab has described) requires continuous submersion of the organoids in a 3D configuration. When plated in 2D (for instance under ALI conditions), organoid-derived cells can be studied but not

further expanded. To the support of our approach: Lungs of newborns are fully functional within minutes, while the airways and alveoli were never exposed to air prior to the moment of birth. This is now more extensively discussed in lines 82-87, 111-113, 115.

In Fig.3, a simple question arises, as to why the PCD organoids are not showing any downregulation of the respective mutated genes in PCD4 and PCD6 lines (CCDC65 and LRRC6).
A: Most mutations in our study are missense mutations which lead to single amino acid changes, which should not lead to Nonsense Mediated Decay. The mutation found in CCDC65 indeed leads to a frameshift. Yet, escape from nonsense-mediated decay is not unusual and has for instance been described for as general phenomenon for multiple genes by (Dyle et al., 2020).

Also, these control versus patient cells are showing up similar red/blue up/down regulated genes - yes, but it is not possible to distinguish the PCD samples from the control - as the two PCD look so dis-similar to each other.
A: We agree that the two PCD patients look so dissimilar which is in line with the general effects of the differentiation protocol. We revised our visualisation of the mRNA sequencing results. Due to donor-to-donor variation, we depicted Log2Fold changes in the initial manuscript. We have now included a batch-effect normalisation to correct for this donor-donor variation. This method was included to depict row z-scores instead of Log2Fold changes in figure 3a,d and 4a. Row z-scores without the donor-effect normalisation are depicted in Appendix Figure S2. This heatmaps shows clustering of 2 samples (PCD1_DNAI2 and Normal2_WT) based on culture method but shows separate clustering of PCD4_CCDC65. Batch effect normalisation allows for visualisation of a general effect of clustering AOs in CilM independent of the origin. To clarify the use of normalised data, more extensive legends are included in figure 3 and 4 and to methods section "RNA isolation and RNA sequencing" which explains the method for normalisation.

These transcriptomic results look hard to decipher for their true value - the huge number of differently expressed genes, 17,000+ genes in S1, is not discussed in any depth (does not give confidence of a meaningful cut-off, unless they have a lot more data generated for more samples and controls than 1).
A: Well taken. Table EV2 showed all genes and not just all differentially expressed genes. We have included an additional table which only includes the 193 differentially expressed genes (130 upregulated and 63 downregulated) (adjusted p-value <0.01 and abs(log2fold) > 1.5). This table is now referenced as well as some additional information of differential expression analysis in line 158-160.

GO annotation has been done for all genes, but then this should be added to the supplementary gene tables S1 and S2 then.
A: Our original text was not clear on this point: GO term analysis was performed on significantly differentially expressed genes with log 2 fold difference of 1.5 and adjusted p-value lower than 0.01. These are the genes that are presented in the heatmap in figure 3a and Appendix Figure S2. This is now better explained in methods and materials section "RNA isolation and RNA sequencing".

In these supplementary tables, column titles are also not separated correctly for Table S1, S2.
A: We thank the reviewer for noting this. The files were saved as comma-separated values files which can lead to miss-alignment of column titles. The files are now uploaded as excel files which resolves the issue raised.

What can we conclude about differences between these samples from this data?
A: Overall a similar process is seen between donors without much differences. As mentioned, there are some differences between donors which can be corrected in mRNA sequencing analysis by the batch effect correction. The differences found in flow cytometry and IHC quantification underline the patient-specificity of the organoid lines. We do, however, consistently observe upregulation of cilia-related genes and downregulation of secretory-related genes in AOs differentiated for 21 days in CilM compared to the same donor AOs in AO medium as well as an increase in cells with cilia. This shows that the differentiation protocol is robust and can be employed independent of the origin of the AO line.

Top 10 GO term enrichment analysis, this is not explained.
A: GO term enrichment analysis is now more extensively explained in methods section "RNA isolation and RNA sequencing" in the revised manuscript. In short, differentially expressed genes were analysed using the analysis tool from the PANTHER Classification System on http://geneontology.org/.

Table S2 is an extracted list of ciliary genes. This has not been properly explained.
A: We have generated this list using published papers describing genes involved in cilium build-up and transport, as also now referenced in the text in line 164.

Fig 4, again it would be reassuring to know that PCD patient cells are viewed as any different from control or not, the 3 lines shown seem quite varied. It is stated that genes upregulated in CilM organoids were characterised as being involved in 'cilium movement' by GO-term analysis and as well as in the GO terms 'ciliated cell functioning including axoneme assembly' and 'cilium assembly' - is this statement fully true, for all three lines displayed, why is data selected for two patients only?
A: We agree that we have only shown two patients and one healthy donor in our bulk mRNA sequencing. The current data however shows that in these 50% of the lines, a general increase in cilia-related genes and downregulation of secretory cell genes can be observed. These differences are observed in each of the three analysed donors. The differentiation protocol can therefore be seen as robust and independent of the AO's origin. Additionally, to answer whether all PCD lines show upregulation of the same transcriptomic profile, we have provided RT-qPCR data of all PCD AOs (Figure EV1B) which shows that all AOs upregulate known cilia-related genes and downregulate secretory cell marker genes when differentiated in CilM for 14 days. Therefore, more extensive analysis of the other lines using bulk mRNA sequencing would be redundant.

What happened to donors PCD1 and PCD2? If other cultures failed this should be documented.
A: We agree that we unfortunately did not extensively explain current nomenclature. hsLung93N was originally named hsLung93N-PCD1 as was hsLung116N-PCD2. The healthy donor lines lost their PCD numbering in

© European Molecular Biology Organization

preparation of the initial manuscript. To create a clear nomenclature of the lines, we have now altered the lines to Normal1_WT (hsLung93N-PCD1) and Normal2_WT (hsLung116N-PCD2), and PCD1_DNAI2 (hsLung105-PCD3), PCD2_LRRC6 (hsLung108-PCD4), PCD3_DNAH11 (hsLung109-PCD5), PCD4_CCDC65 (hsLung111-PCD6). This includes the mutated gene as requested in minor comments.

The characterisation in Fig 5 is quite weak. The only functional evidence of preserved phenotype in PCD is that the mucous doesn't move as it does in normal organoids. No ciliary beat pattern or frequency is given, where is the data? The text has mentioned nNO, but this has not been measured in the organoids. Also no cilia structural analysis is performed on the organoids?
A: We have now included videos of immotile cilia (Video EV4). Structural analysis using TEM has as well been included in the revised manuscript (Figure 5B-H).

Minor

1. Instead of just cell line PCD3,4,5,6 it would be more help if these names could incorporate the relevant PCD gene as well, to make more clear for reading in the text, what is expected in terms of motility and cilia structural defect.
A: As mentioned in earlier major comment, the mutated gene has now been added to the nomenclature of the AO line.

2. Intro mentions respiratory distress due to narrowing of the airway, but bronchiectasis typically is characterised by widening and scarring of the airway.
A: We have changed the text accordingly in line 41 to "impaired oxygen transport".

3. 'waxing and waning' is not a clear description, this needs an edit.
A: The description is changed to "varying severity of the symptoms" in line 78.

4. typo 'relates', 3rd line of Results.
A: We thank the reviewer for noticing the typo. It has been corrected in line 99.

5. Ensure correct variant annotation throughout. This needs to mention transcript e.g. CCDC65 NM_033124.4:c.877_878delAT (any gene symbols in italics).
A: Correct variant annotation has been added to figure 1A. All genes have been changed to italic.

Referee #3:

Summary
This research paper by van der Vaart et al entitled "Modelling of Primary Ciliary Dyskinesia using Patient-derived Airway Organoids" demonstrates the utility of airway organoids (AO) derived from nasal brushings as a cell-based model that can be used to study PCD and potentially find new therapies. Here, the authors described a method to generate AO from cells isolated from nasal epithelia. These

AO are expandable when cultured in "expansion media" (AO media) and further differentiate into ciliated cell lineages in media containing BMP4 and DAPT (CilM). This is demonstrated through SiR-Tubulin live imaging of "beating" cilia and staining for acetylated alpha-tubulin. Importantly, AOs can be generated from nasal brushings from individuals harboring mutations affecting motile cilia function or assembly (PCD3-6). Live imaging of cilia from PCD AO shows disrupted function. Finally, the authors perform bulk sequencing of the AO in AO media versus CilM to show that CilM supports differentiation of the cell to ciliate cells at the expense of secretory cell lineages.

Tissue-specific organoids have become an increasingly important model to study development and pathological causes of disease. PCD, a pulmonary disease caused by dysfunctional motile cilia leading to impaired mucociliary transport of foreign objects has been linked to genetic mutations, of which there are now >40 disease causing mutations. Methods to diagnose PCD are limited and I agree with the authors that there is a need to develop cell-based models for diagnosis and therapy discovery. While this paper does contain some convincing data to support the utility of the AO to model PCD as mentioned above, my enthusiasm for this work is dampened by several major factors: The novelty of the work is lacking, additional validation of the model is needed, and the claim that AO can be used for PCD diagnostics is a big stretch, at least with the current methods shown here. There is not sufficient data presented to support that the model is truly reflective of the airway phenotype let alone it can be used to model PCD airway disease. For this paper to be accepted, significant additions are needed with careful detailed analyses of the model.

Major weaknesses:

1) Novelty of the work - Developing AO from nasal brushings is not new, neither is the differentiation of the AO to ciliated cells using the sample factors described in the protocol. Perhaps the novelty here is the demonstration that AO can be used to model PCD but this leads to the second major point.

A: We agree that the development of AOs from nasal brushings is not novel . The differentiation of ciliated cells in AOs using a defined medium without the need of feeder cells is, however, novel. Current studies rely either on the spontaneous differentiation of ciliated cells in 3D airway spheres in which differentiation is very limited (Hynds et al., 2019; Sachs et al., 2019) *or on undefined commercial media like PneumaCult-ALI medium of Stem Cell Technologies* (Zhou et al., 2018). *The novelty remains in the use of defined medium. We agree that activating BMP signals and inhibition of NOTCH pathway to induce ciliated cell differentiation has been described before, but not yet in* in vitro *adult stem cell cultures (such as AOs). This novelty is now more extensively explained in the introduction (line 89-93) and discussion (line 254-277)*

2) The need for additional validation of the model. While the authors provide some characterization of their AO model with bulk sequencing and staining for cilia, additional validation especially of the mutant phenotypes should be done. The data as is, is not sufficient to convince me that the model is appropriate and can truly be

© European Molecular Biology Organization

used in understanding PCD pathogenesis/diagnosis.
I. Specific staining for dynein proteins is needed to demonstrate genetic-phenotype effects of the mutations and how the phenotype impacts the axoneme.
A: We have included additional patient-specific data: TEM images of axonemes of PCD2_LRRC6 (hsLung108-PCD4 in previous manuscript and PCD3_DNAH11 (hsLung109-PCD5) (figure 5c-h) and live imaging of ciliary immobility in PCD2_LRRC6 (Video EV4).

II. Demonstrate axoneme impairment with electron micrographs and functional analyses of the impaired motile cilia.
A: We have included additional data of ultrastructural changes in the axoneme in figure 5c-h. This clearly discriminates ultrastructural abnormalities in PCD2_LRRC6 and PCD3_DNAH11. These impairments are explained in line 192-203.

III. Snapshot images of SiR-Tubulin stained cilia to demonstrate "immotile cilia" is not convincing. Can the authors use other assays to measure motility? Ie. Mapping fluorescent bead movement?
A: The 3D architecture of the organoids lets the fluorescent beads flow in multiple dimensions which include the z-plane of the microscope. We have therefore chosen an alternative approach: SiR-Tubulin live-stain videos are now given in Video EV4. This video demonstrates the immobility of cilia in differentiated PCD-derived AO.

IV. Can editing the mutation in these AO restore motile cilia function? This might be an indirect method to validate their model.
A: See reviewer 1. Due to the nature of the mutations, we applied the newly developed prime editing CRISPR/Cas9 tool (Anzalone et al., 2019; Geurts et al., 2020; Schene et al., 2020) *to repair the* DNAH11 *mutation in PCD3_DNAH11.*

3) The rationale for differentiation of the AO is unclear. Manipulating the Notch and BMP pathways have previously been shown to induce ciliated cell differentiation and the authors use these approaches to "increase" ciliated cell differentiation in the AO. It is unclear to me what is the rationale for doing so? Is it to increase the number of ciliated cells to monitor function (ie beating)? As a future goal to model patient-specific responses or for therapy discovery, does manipulating the cell type in the AO truly reflective of PCD disease?
A: Since PCD is characterised as a disease of ciliated cells, the differentiation helps identifying small differences that had been overlooked if ciliated cell numbers were limited. To clarify the need for differentiation, we have included additional text in the results section (line 123).

I. The bulk sequencing data in Figure 3 appears to be missing half of the data. Specifically, both figure legend and in the results section of the paper, the authors described a difference in gene expression of AO in AO media versus CilM media for each cell line tested and yet it is unclear what the heatmap is showing other than 3 columns representing the 3 cell lines with what appears to be similar expression patterns of genes for AO or CilM? One cannot evaluate the data if the data is not there.
A: Well taken. Initially, the heatmaps shown in figure 3a,d and 4a showed log2 fold changes of AOs in CilM compared to AOs of the same donor in AO medium. This

normalisation would create three columns with all genes represented as 1 for AO medium samples next to the shown three columns of CilM. To save space, these normalised columns were removed and only CilM columns are depicted. This normalisation was required since there are differences in absolute gene counts per donor (now indicated in Appendix Figure S2). We have included additional batch effect correction to remove donor-donor variation. These corrected counts are plotted as row z-scores in revised figure 3a,d and figure 4a. Each legend specifies whether donor-corrected counts or uncorrected DESeq2 normalised counts are depicted. This method of normalisation is more extensively explained in line 166-168 and methods section "RNA isolation and RNA sequencing". In figure 3 we hope to convey a general transcriptomic change induced by CilM, independent of the donor.

II. Immunostaining, at the very least, is needed to validate the lineages suggested by gene expression. It is hard to know whether a uniform homogenous AO population is formed or there are heterogenous AOs where differences in cell compositions and if so, how does this affect modelling of PCD?
A: Immunostainings were added of the basal cell layer. This shows no clear differences in cell number of distribution of the basal cells. We therefore believe that CilM mostly induces transcriptomic changes in these basal cells. The AOs are comprised of a KRT5 basal layer. We could identify secretory cells by markers of SCGB1A1 in AO medium AOs and secretory-like cells in TEM images of AOs in AO medium but never in CilM AOs. These changes in cellular composition seen by immunofluorescence have been included in figure 4e-f and described in text in line 175-181.

III. While using nasal brushings may be good surrogates of the airway epithelium, the authors should present data to support this. It is unclear if nasal epithelia-derived organoids can model airway epithelial responses.
A) Well taken. While we agree that nasal epithelium might not be similar to airway epithelium, the use of nasal epithelium as surrogate for PCD disease diagnosis and modelling dates back to 1991 (Barlocco et al., 1991). *Multiple studies show PCD hallmarks in nasal epithelium* (Bricmont et al., 2020; Coles et al., 2020; Hirst et al., 2010; Lucas et al., 2014; Marthin et al., 2017; Shoemark et al., 2017). *Recently, a meta-analysis showed that there are no significant differences between nasal brushings and tracheobronchial biopsies in terms of diagnostic measures* (Adil et al., 2017). *We therefore believe non-invasively received nasal biopsies are preferred over airway biopsies that require general anaesthesia. The use of nasal samples as airway surrogates has been included in the results section (line 101).*

IV. Clarify the novelty of the differentiation protocol and how it relates to previous publications using similar media components to generate ciliated cells.
A: The differentiation of ciliated cells in AOs using a defined medium without the need of feeder cells is novel. Current studies rely either on the spontaneous differentiation of ciliated cells in 3D airway spheres in which differentiation is very limited (Hynds et al., 2019; Sachs et al., 2019) *or on undefined commercial media like PneumaCult-ALI medium of Stem Cell Technologies* (Zhou et al., 2018).

Minor weaknesses:
1) This essentially is an n=1 study for each cell line from 1 nasal brushing sample

for each mutation and control. Cell composition and cell number will change with each nasal brushing. Are these results reproducible with additional brushings. Similarly, are these results reproducible with additional nasal brushings from other individuals with the same mutation?
A: We feel that the fact that we observe phenotypic abnormalities of cilia in organoid lines of individual patients with ciliopathies (but never in healthy controls) validates our approach. Other individuals with similar mutations would not represent a genetic duplicate (given genetic background differences). This issue could potentially be resolved with taken multiple nasal brushings from the same patient. Ethically this would raise concerns. Nevertheless, the generation of organoids depends on the presence of multiple stem cells in the brushing; this dilutes out the effect of phenotypes of individual stem cells.

2) Authors claim that the AOs can be expanded for >1 year (stated in the discussion). Where is the data to support this claim? And does the phenotype (cell composition, function) of the AOs change with serial passaging/freeze-thaw? Does the karyotype change over time? I am not convinced that primary cells have the capacity for such long-term serial passaging and culture without entering senescence or acquiring oncogenic mutations. The authors should carefully characterise the "long-term" AOs and present this data.
A: The referee may not be familiar with adult-stem cell based organoid technology. There are now hundreds of studies that exploit adult stem cell-based organoid technology for the extensive expansion and manipulation of cells derived from multiple different human organs. Indeed, such organoids are stable over years in terms of growth characteristics, karyotype, telomere length and phenotype. They can be subcloned, CRISPR-ed, frozen/thawed etc. while remaining entirely stable. Long-term stability of AOs (>1 year of continuous culture) has been demonstrated conclusively by Sachs and colleagues (Sachs et al., 2019)*. In line, the AOs generated for this manuscript have been cultured for >35 passages. Key to this technology is the use of potent Wnt receptor ligands. Indeed, senescence, telomere shortening etc does not occur in these cultures when growth factor conditions are optimized. Of note, the same phenomena do not take place in vivo over time periods of years (rather than over multiple decades). From this, one can only conclude that senescence is largely an artefact of inadequate culture conditions.*

3) Figures 2A, 2D and 2E are showing the same thing...cilia in the AO. This is not an efficient use of space as it seems redundant. Instead the authors should consider providing additional characterization of the ciliated AO. Ie. Data to show that AO after >1 year maintains the phenotype of early AO.
A: Well taken. We have used the space to add new data of PCD AOs.

4) Quantification of the number of ciliated cells and other cell types by FACS is a better measure than acetylated tubulin positively over surface area (Figure 2B).
A: We agree that FACS data might better quantify ciliated cell numbers. We have included flow cytometry data to validate our findings in Figure EV2.

5) Showing presence of cilia in Figure 5 in AO from PCD patients does not indicate "PCD phenotypes". Additional data is needed here to support this.
A: We agree that the title of the figure 5 was not representative of the data shown. The legend title has been changed to be in line with the data presented. Figure title

now states "PCD AOs differentiated towards ciliated cells allow live and nanoscopic analysis".

6) The authors make a statement in the discussion "...we describe a long-term disease modelling strategy using AOs...". How does the present data support this statement?
A: Well taken, as mentioned in our discussion of reviewers minor point 2, the AOs have been shown to be culturable for long term. Additionally, the PCD-patient-derived AOs were cultured in our hand for over 35 passages (over one year). This line has been included in the manuscript (line 114-115).

7) The statement "The AO approach can therefore be seen as an additional and potentially definitive diagnostic assay of PCD patients, abolishing the need, in some cases, for multiple inconclusive diagnostic tests." is a bit of a stretch. This needs to be revised.
A: Agreed. This has therefore been revised in the manuscript to "The AO approach can therefore be seen as an additional and potentially definitive diagnostic assay of PCD patients".

1st Revision - Editorial Decision 16th September 2021

Dear Hans,

Thank you for the submission of your revised manuscript. We have now received the enclosed reports from the referees. Referees 2 and 3 still have some more minor suggestions that I would like you to address and incorporate before we can proceed with the official acceptance of your manuscript. Please co-submit a point-by-point response to the referee comments for faster evaluation.

A few other editorial requests also need to be addressed:

- If you show the same image in 2 different figures please explain this in the figure legends.

- Please correct the reference format to the EMBO reports style (same as Harvard style).

- The Funding info should be part of the Acknowledgement section.

- Fig 2F callout is missing. Fig EV2 panel callouts are missing. Appendix Fig S1 panel callouts are missing. Table EV4 callout is missing. Please add.

- Please submit the movies as zipped files (one zip file per movie) together with their legends.

- Please rephrase "Competing interest" to "Conflict of Interest"

- The tables in the method section need to be called Table 1, Table 2 and Table 3, and a title should be added to each table. The tables also need to be moved to the end of the manuscript file.

- Please remove the movie and EV table legends from the manuscript file. And remove the figure legends from the figure files.

- I attach to this email a related manuscript files with comments by our data editors. Please address all comments in the final manuscript.

EMBO press papers are accompanied online by A) a short (1-2 sentences) summary of the findings and their significance, B) 2-3 bullet points highlighting key results and C) a synopsis image that is exactly 550 pixels wide and 200-600 pixels high (the height is variable). You can either show a model or key data in the synopsis image. Please note that text needs to be readable at the final size. Please send us this information along with the revised manuscript.

I finally would like to suggest a few minor changes to the abstract that needs to be written in present tense:

Patient-derived human organoids can be used to model a variety of diseases. Recently, we described conditions for long-term expansion of human airway organoids (AOs) directly from healthy individuals and patients. Here, we first optimize differentiation of AOs towards ciliated cells. After differentiation of the AOs towards ciliated cells, these can be studied for weeks. When returned to expansion conditions, the organoids readily resume their growth. We apply this condition to AOs established from nasal inferior turbinate brush samples of patients suffering from primary ciliary dyskinesia (PCD), a pulmonary disease caused by dysfunction of the motile cilia in the airways. Patient-specific differences in ciliary beating are observed and are in agreement with the patients' genetic mutations. More detailed organoid ciliary phenotypes can thus be documented in addition [OK?] to the standard diagnostic procedure. Additionally, using genetic editing tools, we show that a patient-specific mutation can be repaired. This study demonstrates the utility of organoid technology for investigating hereditary airway diseases such as PCD.

Please let me know whether you agree with these changes.

I look forward to seeing a new revised version of your manuscript as soon as possible. Please use this link to submit your revision:
https://embor.msubmit.net/cgi-bin/main.plex

Best wishes,
Esther

Esther Schnapp, PhD
Senior Editor
EMBO reports

Referee #1:

The revised work addresses all my concerns and the authors have significantly improved the the manuscript. Congratulations and I applaud the authors for bringing new technical advances to the field!
One minor comment: Video file in EV5 seem to have some encoding errors. I suggest you fix these problems.

Referee #2:

In this re-review, I am generally happy for this paper to be accepted, it looks much better.

Just one issue left - the TEM description for Figure 5 is currently not correct. In TEM, DNAH11=normal TEM structure and LRRC6= inner and outer dynein arms defects only.
So,
PCD3_DNAH11, mutations in this gene do not affect inner arms. DNAH11 is an

outer dynein arm component, this is a mistake in TEM interpretation, the inner arms are notoriously challenging to image and often look present/absent, have they counted 300 cross sections and looked at the % inner arms affected? Probably not:
- DNAH11 would not show up any defect in conventional TEM, this is well known.

PCD2_LRRC6, the 'ruffled edge' in the TEM is not likely relevant for comment, this is a feature seen all the time in TEM, unless authors have done some kind of survey where they can state this is a notable defect? - it is better not to mention. Similarly the dynein arms would both be affected in LRRC6 deficient cells yes, but not any radial spoke disturbance, again better take reference to RS out of the text, unless there is really evidence that this was found at significant levels, because these kinds of rare artefacts are often observed in cilia TEM edven in healthy people.
Challenging TEM in PCD does require expertise - if these wrong statements about the TEM are left in the text, then it is not looking accurate but mistaken.

Two typos to correct:
Line 148 '...cell numbers in all PCD AOs was observed after 14 days...'
Line 248 homozygous and heterozygous repair'.

Referee #3:

The authors have made considerable additions based on the reviewer's comments and the manuscript is significantly improved with much more clarity in the rationale, experimental design and readouts. There remains some strong, over-reaching statements and concerns that in my opinion should be revised.
First, I appreciate that the authors responded to several of the reviewer's question about gene editing the AO as a potential cell-based transplantation therapy for PCD. However, the outcome of the editing (albeit due to technical issues) did not yield a reliable edited source of AO to determine whether gene correction translated to functional correction which now weakens the paper. Moreover, technically the AO itself was not gene corrected. If the AOs had to be dissociated into single cells prior to electroporation for gene editing to occur, this approach is no different than dissociated 2D cultures for electroporation, especially since the AOs could not be expanded therafter. This raises the original concern of how novel is the work in relation to it being a model of PCD to test for therapies etc. Along a similar train of thought, what would be the benefit of generating a "long-term" patient-derived model here? Needs a better justification here.
The authors would argue that they've created AO with improved ciliated cell generation that can be used as a diagnostic tool for PCD (Line 231-236). That I find is the issue and too strong of a conclusion based on 4 PCD lines, 2 "wild-type" lines. Were these studies performed in a blinded, unbiased manner? This was not clear in the methods or results.
The authors alluded to difficulties in current diagnostic tools of PCD because the phenotype can be subtle or complex (Line 61-68). How then, does motile cilia movement be any better at predicting/diagnosing PCD given in-vitro ciliary movement may be affected by many extraneous factors (ie. prolonged exposure to room temperature) not related to the disease? In my opinion, creating an in vitro

model of MCC defect to study molecular mechanisms driving MCC impairment is sufficient to warrant publication. No need to justify with "diagnostics" or "therapy" which is beyond the current status of the work.
There are issues with the gene expression sequencing results in Fig 4A for PCD1 which looks completely different in Fig EV4. Are these different sequencing results from different batches of differentiation? If so then why the opposite results? and if that is the case, how reliable is the differentiation/AO?
Duplicating images in main figure and supplementary figure should be avoided.
And finally, it is unclear how AO grown in CiM media comprised mainly of basal and ciliated cells can not self-renew as abundantly as AO in traditional media which is comprised on mainly secretory cells (Figure 4A is strikingly contrasting the AOs in different culture media). It is traditionally believed that basal stem cell (TP63+KRT5+) are the cells that enable self-renewal of organoids and yet the gene expression here seems to suggest another cell type is contributing to AO self-renewal in AO media, which is surprising.

Having said that, should the authors carefully factor in these concerns on a revised version, it would be a stronger manuscript for publication consideration.

2nd Authors' Response to Reviewers 19th September 2021

Dear Editor
We respectfully resubmit our manuscript entitled "Modelling of Primary Ciliary Dyskinesia using Patient-derived Airway Organoids" by Jelte van der Vaart et al. We believe to have addressed all editorial and referees' comments.
Below, we give a point-by-point rebuttal. Changes in the manuscript have been indicated in green.
We hope that -with these changes- you will be able to accept our manuscript for publication
With kind regards, on behalf of all authors
Hans Clevers

Editorial requests
E: If you show the same image in 2 different figures please explain this in the figure legends.
A: This reference to the same image in 2 different figures has been added to the legends.

E: Please correct the reference format to the EMBO reports style (same as Harvard style).
A: Reference format has been changed to Harvard style.

E: The Funding info should be part of the Acknowledgement section.
A: We've moved the Funding info to the Acknowledgement section.

E: Fig 2F callout is missing. Fig EV2 panel callouts are missing. Appendix Fig S1 panel callouts are missing. Table EV4 callout is missing. Please add.
A: *Mentioned callouts have now been added to the revised manuscript. Figure 2f in line 170. Fig EV2 panels in line 164-165. Appendix Fig S1 panels in line 135. Table EV4 in line 183.*

E: Please submit the movies as zipped files (one zip file per movie) together with their legends.
A: Movies will be uploaded as a zip file per movie including their legend

E: Please rephrase "Competing interest" to "Conflict of Interest"
A: Section header has been changed to Conflict of Interest

E: The tables in the method section need to be called Table 1, Table 2 and Table 3, and a title should be added to each table. The tables also need to be moved to the end of the manuscript file.
A: Point taken. We have moved all tables from the method section to the end of the manuscript and named them accordingly.

E: Please remove the movie and EV table legends from the manuscript file.
A: Movie and EV table legends have been removed from the manuscript file

E: And remove the figure legends from the figure files.
A: Figure legends have been removed from the figure files

E: I attach to this email a related manuscript files with comments by our data editors. Please address all comments in the final manuscript.
A: The comments raised by the data editors have been added to the figure legends

E: EMBO press papers are accompanied online by A) a short (1-2 sentences) summary of the findings and their significance, B) 2-3 bullet points highlighting key results and C) a synopsis image that is exactly 550 pixels wide and 200-600 pixels high (the height is variable). You can either show a model or key data in the synopsis image. Please note that text needs to be readable at the final size. Please send us this information along with the revised manuscript.
A: We have included a short summary and bullet points to the revised manuscript. A synopsis image is created and uploaded along with the revised manuscript

E: I finally would like to suggest a few minor changes to the abstract that needs to be written in present tense.
A: We agree with the adjusted abstract and have replaced this in our revised manuscript file

Referee #1:

R: The revised work addresses all my concerns and the authors have significantly improved the the manuscript. Congratulations and I applaud the authors for bringing new technical advances to the field!
One minor comment: Video file in EV5 seem to have some encoding errors. I suggest you fix these problems.
A: We thank the referee for this. We have looked into Video EV5 and fixed the encoding error.

Referee #2:

In this re-review, I am generally happy for this paper to be accepted, it looks much better.

R: Just one issue left - the TEM description for Figure 5 is currently not correct. In TEM, DNAH11=normal TEM structure and LRRC6= inner and outer dynein arms defects only.
So, PCD3_DNAH11, mutations in this gene do not affect inner arms. DNAH11 is an outer dynein arm component, this is a mistake in TEM interpretation, the inner arms are notoriously challenging to image and often look present/absent, have they counted 300 cross sections and looked at the % inner arms affected? Probably not: - DNAH11 would not show up any defect in conventional TEM, this is well known.
A: We agree with the referee that DNAH11 mutations have not been described to lead to loss of inner dynein arms. The analysed was performed on approximately 10-20 cross sections. We have added an additional line in the electron microscopy

section to remove the statement of DNAH11 mutations leading to loss of inner dynein arms.

PCD2_LRRC6, the 'ruffled edge' in the TEM is not likely relevant for comment, this is a feature seen all the time in TEM, unless authors have done some kind of survey where they can state this is a notable defect? - it is better not to mention. Similarly the dynein arms would both be affected in LRRC6 deficient cells yes, but not any radial spoke disturbance, again better take reference to RS out of the text, unless there is really evidence that this was found at significant levels, because these kinds of rare artefacts are often observed in cilia TEM edven in healthy people.
A: We agree that ruffled edges and radial spoke disturbances in TEM might not be relevant as patient parameter. Therefore, we have removed the observation from the manuscript text.

R: Two typos to correct:
Line 148 '...cell numbers in all PCD AOs was observed after 14 days...'
A: We thank the referee for noticing the typo and we have corrected this.

R: Line 248 homozygous and heterozygous repair'.
A: We thank the referee for noticing the typo and we have corrected this.

Referee #3:

R: The authors have made considerable additions based on the reviewer's comments and the manuscript is significantly improved with much more clarity in the rationale, experimental design and readouts. There remains some strong, over-reaching statements and concerns that in my opinion should be revised.
First, I appreciate that the authors responded to several of the reviewer's question about gene editing the AO as a potential cell-based transplantation therapy for PCD. However, the outcome of the editing (albeit due to technical issues) did not yield a reliable edited source of AO to determine whether gene correction translated to functional correction which now weakens the paper. Moreover, technically the AO itself was not gene corrected. If the AOs had to be dissociated into single cells prior to electroporation for gene editing to occur, this approach is no different than dissociated 2D cultures for electroporation, especially since the AOs could not be expanded thereafter. This raises the original concern of how novel is the work in relation to it being a model of PCD to test for therapies etc. Along a similar train of thought, what would be the benefit of generating a "long-term" patient-derived model here? Needs a better justification here.
A: Point taken. While we agree that the potential of AOs as transplantation material would be greatly improved if functional assays could be performed on gene corrected AOs, the existing technology does not allow for outgrowth of such corrected organoid lines. The advantage of patient-derived long-term models however still stands in our opinion. In contrast to 2D cell cultures, AOs can be expanded for extensive periods of time and can be generated from small biopsies. This allows for extensive expansion of patient-specific material which (when outgrowth of electroporated AOs will be optimised) can not only be transplanted but can be studied in great detail. The expansion capacity of the AOs allows for

years of studies of the same patient material without the need for additional biopsies. This was already all clarified in the text.

R: The authors would argue that they've created AO with improved ciliated cell generation that can be used as a diagnostic tool for PCD (Line 231-236). That I find is the issue and too strong of a conclusion based on 4 PCD lines, 2 "wild-type" lines. Were these studies performed in a blinded, unbiased manner? This was not clear in the methods or results.
A: All quantifications were performed in a blinded manner (this is now clearly stated). We agree that our claim of increased diagnostic measure is based on only 4 lines. We have added a statement in line 252: "Additional patient-derived lines will be needed to further strengthen this conclusion.".

The authors alluded to difficulties in current diagnostic tools of PCD because the phenotype can be subtle or complex (Line 61-68). How then, does motile cilia movement be any better at predicting/diagnosing PCD given in-vitro ciliary movement may be affected by many extraneous factors (ie. prolonged exposure to room temperature) not related to the disease? In my opinion, creating an in vitro model of MCC defect to study molecular mechanisms driving MCC impairment is sufficient to warrant publication. No need to justify with "diagnostics" or "therapy" which is beyond the current status of the work.
A: We agree that the AOs are most valuable as tools to study the molecular mechanisms of ciliary function/movement and MCC. The extraneous factors of in vitro cultures can indeed impair the cilia movement. However, we have always compared the parameters to healthy control AOs which are subject to similar extraneous factors. The translation of the in vitro AO culture to patients remains a promise, but we have shown in other organoid systems as well as in AOs that organoid responses predict patient responses very well (Berkers et al, 2019; Driehuis et al, 2019a, 2019b; Lee et al, 2018; Tiriac et al, 2018; de Winter-De Groot et al, 2018). This does show promise for PCD AOs as well. We would therefore like to stay with our statement on this issue.

R: There are issues with the gene expression sequencing results in Fig 4A for PCD1 which looks completely different in Fig EV4. Are these different sequencing results from different batches of differentiation? If so then why the opposite results? and if that is the case, how reliable is the differentiation/AO?
A: We agree that the figures might appear contradictory. The legends however explain the differences between the heatmaps. While figure 4A shows row z-scores of donor-corrected normalised counts (comparing CilM to AO medium within the same donor), figure EV4 shows row z-scores of non-corrected Log2 transformed normalised counts (normalised over all samples and not within one donor). Thus, in both graphs a similar trend is visualised (increase in ciliated cell markers and loss of secretory cell markers)..

And finally, it is unclear how AO grown in CiM media comprised mainly of basal and ciliated cells can not self-renew as abundantly as AO in traditional media which is comprised on mainly secretory cells (Figure 4A is strikingly contrasting the AOs in different culture media). It is traditionally believed that basal stem cell (TP63+KRT5+) are the cells that enable self-renewal of organoids and yet the gene

expression here seems to suggest another cell type is contributing to AO self-renewal in AO media, which is surprising.
A: Point taken. Basal cells are indeed viewed as the principle stem cells of the airways. Yet, Club cells are well known to perform the same function (Zheng *et al*, 2017; Kathiriya *et al*, 2020; Reynolds & Malkinson, 2010; Shafiquzzaman *et al*, 2020)*. Moreover, there have been reports of secretory cells dedifferentiating into basal cells upon damage (Tata et al, 2013). We agree that since the KRT5+ basal cell population seems unchanged, the capacity of outgrowth could be similar. Therefore, we propose that the KRT5-negative cells in expansion conditions are (immature) differentiating cells (most likely secretory but could also be ciliated cells) which convert to organoid-forming cells when placed in expansion conditions compared to the mature ciliated cells in differentiation condition.*

2nd Revision - Editorial Decision 21st September 2021

Dear Hans,

I am very pleased to accept your manuscript for publication in the next available issue of EMBO reports. Thank you for your contribution to our journal.

Given that you as corresponding author are in the Netherlands, you are probably eligible for publication of your article in the open access format in a way that is free of charge for the authors. Please contact either the administration at your institution or our publishers at Wiley (emboreports@wiley.com) for further questions. The general information of Wiley's agreements with the Netherlands can be found here: https://authorservices.wiley.com/author-resources/Journal-Authors/open-access/affiliation-policies-payments/vsnu-agreement.html

At the end of this email I include important information about how to proceed. Please ensure that you take the time to read the information and complete and return the necessary forms to allow us to publish your manuscript as quickly as possible.

As part of the EMBO publication's Transparent Editorial Process, EMBO reports publishes online a Review Process File to accompany accepted manuscripts. As you are aware, this File will be published in conjunction with your paper and will include the referee reports, your point-by-point response and all pertinent correspondence relating to the manuscript.

If you do NOT want this File to be published, please inform the editorial office within 2 days, if you have not done so already, otherwise the File will be published by default [contact: emboreports@embo.org]. If you do opt out, the Review Process File link will point to the following statement: "No Review Process File is available with this article, as the authors have chosen not to make the review process public in this case."

Thank you again for your contribution to EMBO reports and congratulations on a successful publication. Please consider us again in the future for your most exciting work.

Best regards,
Esther

Esther Schnapp, PhD
Senior Editor
EMBO reports

EMBO PRESS

YOU MUST COMPLETE ALL CELLS WITH A PINK BACKGROUND ↓

PLEASE NOTE THAT THIS CHECKLIST WILL BE PUBLISHED ALONGSIDE YOUR PAPER

Corresponding Author Name: Hans Clevers
Journal Submitted to: EMBO Reports
Manuscript Number: EMBOR-2020-52058

Reporting Checklist For Life Sciences Articles (Rev. June 2017)

This checklist is used to ensure good reporting standards and to improve the reproducibility of published results. These guidelines are consistent with the Principles and Guidelines for Reporting Preclinical Research issued by the NIH in 2014. Please follow the journal's authorship guidelines in preparing your manuscript.

A- Figures
1. Data
The data shown in figures should satisfy the following conditions:

- the data were obtained and processed according to the field's best practice and are presented to reflect the results of the experiments in an accurate and unbiased manner.
- figure panels include only data points, measurements or observations that can be compared to each other in a scientifically meaningful way.
- graphs include clearly labeled error bars for independent experiments and sample sizes. Unless justified, error bars should not be shown for technical replicates.
- if n< 5, the individual data points from each experiment should be plotted and any statistical test employed should be justified
- Source Data should be included to report the data underlying graphs. Please follow the guidelines set out in the author ship guidelines on Data Presentation.

2. Captions

Each figure caption should contain the following information, for each panel where they are relevant:

- a specification of the experimental system investigated (eg cell line, species name).
- the assay(s) and method(s) used to carry out the reported observations and measurements
- an explicit mention of the biological and chemical entity(ies) that are being measured.
- an explicit mention of the biological and chemical entity(ies) that are altered/varied/perturbed in a controlled manner.

- the exact sample size (n) for each experimental group/condition, given as a number, not a range;
- a description of the sample collection allowing the reader to understand whether the samples represent technical or biological replicates (including how many animals, litters, cultures, etc.).
- a statement of how many times the experiment shown was independently replicated in the laboratory.
- definitions of statistical methods and measures:
  - common tests, such as t-test (please specify whether paired vs. unpaired), simple $\chi 2$ tests, Wilcoxon and Mann-Whitney tests, can be unambiguously identified by name only, but more complex techniques should be described in the methods section;
  - are tests one-sided or two-sided?
  - are there adjustments for multiple comparisons?
  - exact statistical test results, e.g., P values = x but not P values < x;
  - definition of 'center values' as median or average;
  - definition of error bars as s.d. or s.e.m.

Any descriptions too long for the figure legend should be included in the methods section and/or with the source data.

In the pink boxes below, please ensure that the answers to the following questions are reported in the manuscript itself. Every question should be answered. If the question is not relevant to your research, please write NA (non applicable). We encourage you to include a specific subsection in the methods section for statistics, reagents, animal models and human subjects.

USEFUL LINKS FOR COMPLETING THIS FORM

http://www.antibodypedia.com
http://1degreebio.org
http://www.equator-network.org/reporting-guidelines/improving-bioscience-research-repor
http://grants.nih.gov/grants/olaw/olaw.htm
http://www.mrc.ac.uk/Ourresearch/Ethicsresearchguidance/Useofanimals/index.htm
http://ClinicalTrials.gov
http://www.consort-statement.org
http://www.consort-statement.org/checklists/view/32-consort/66-title
http://www.equator-network.org/reporting-guidelines/reporting-recommendations-for-tumo
http://datadryad.org
http://figshare.com
http://www.ncbi.nlm.nih.gov/gap
http://www.ebi.ac.uk/ega
http://biomodels.net/
http://biomodels.net/miriam/
http://jjj.biochem.sun.ac.za
https://osp.od.nih.gov/biosafety-biosecurity-and-emerging-biotechnology/
http://www.selectagents.gov/

B- Statistics and general methods

Please fill out these boxes ↓ (Do not worry if you cannot see all your text once you press return)

1.a. How was the sample size chosen to ensure adequate power to detect a pre-specified effect size?	Sample size was limited due to limited donors. Maximum sample size was used for each experiment.
1.b. For animal studies, include a statement about sample size estimate even if no statistical methods were used.	n.a.
2. Describe inclusion/exclusion criteria if samples or animals were excluded from the analysis. Were the criteria pre-established?	Inclusion criteria for patient samples was the diagnosis of Primary Ciliary Dyskinesia
3. Were any steps taken to minimize the effects of subjective bias when allocating animals/samples to treatment (e.g. randomization procedure)? If yes, please describe.	For quantification of differentiation efficiency based on IHC and flow cytometry, samples were randomly assigned a number. Results were only coupled to donor and treatment after data acquirement.
For animal studies, include a statement about randomization even if no randomization was used.	n.a.
4.a. Were any steps taken to minimize the effects of subjective bias during group allocation or/and when assessing results (e.g. blinding of the investigator)? If yes please describe.	For quantification of differentiation efficiency based on IHC and flow cytometry, samples were randomly assigned a number. Results were only coupled to donor and treatment after data acquirement.
4.b. For animal studies, include a statement about blinding even if no blinding was done	n.a.
5. For every figure, are statistical tests justified as appropriate?	Yes
Do the data meet the assumptions of the tests (e.g., normal distribution)? Describe any methods used to assess it.	Bulk mRNA sequencing quality control was performed using our in-house RNA analysis pipeline v2.1.0 (https://github.com/UMCUGenetics/RNASeq), based on best practices guidelines (https://software.broadinstitute.org/gatk/documentation/article.php?id=3891).
Is there an estimate of variation within each group of data?	Yes

Is the variance similar between the groups that are being statistically compared?	Yes

C- Reagents

6. To show that antibodies were profiled for use in the system under study (assay and species), provide a citation, catalog number and/or clone number, supplementary information or reference to an antibody validation profile. e.g., Antibodypedia (see link list at top right), 1DegreeBio (see link list at top right).	acetylated-α-tubulin (Santa Cruz; sc-23950)<br>actin (Sigma-Aldrich; A5228)<br>SCGB1A1 (Santa Cruz; sc-9773)<br>keratin 5 (Covance; PRB 160P-100)
7. Identify the source of cell lines and report if they were recently authenticated (e.g., by STR profiling) and tested for mycoplasma contamination.	All cell lines were generated at the start of the study and regularly checked for mycoplasma contamination. All tests remained negative.

* for all hyperlinks, please see the table at the top right of the document

D- Animal Models

8. Report species, strain, gender, age of animals and genetic modification status where applicable. Please detail housing and husbandry conditions and the source of animals.	n.a.
9. For experiments involving live vertebrates, include a statement of compliance with ethical regulations and identify the committee(s) approving the experiments.	n.a.
10. We recommend consulting the ARRIVE guidelines (see link list at top right) (PLoS Biol. 8(6), e1000412, 2010) to ensure that other relevant aspects of animal studies are adequately reported. See author guidelines, under 'Reporting Guidelines'. See also: NIH (see link list at top right) and MRC (see link list at top right) recommendations. Please confirm compliance.	n.a.

E- Human Subjects

11. Identify the committee(s) approving the study protocol.	The study was approved by the ethical committee and was in accordance with the Declaration of Helsinki and according to Israeli law under IRB approval number 075-16 HMO. This study is compliant with all relevant ethical regulations regarding research involving human participants.
12. Include a statement confirming that informed consent was obtained from all subjects and that the experiments conformed to the principles set out in the WMA Declaration of Helsinki and the Department of Health and Human Services Belmont Report.	The study was approved by the ethical committee and was in accordance with the Declaration of Helsinki and according to Israeli law under IRB approval number 075-16 HMO. This study is compliant with all relevant ethical regulations regarding research involving human participants.
13. For publication of patient photos, include a statement confirming that consent to publish was obtained.	n.a.
14. Report any restrictions on the availability (and/or on the use) of human data or samples.	n.a.
15. Report the clinical trial registration number (at ClinicalTrials.gov or equivalent), where applicable.	n.a.
16. For phase II and III randomized controlled trials, please refer to the CONSORT flow diagram (see link list at top right) and submit the CONSORT checklist (see link list at top right) with your submission. See author guidelines, under 'Reporting Guidelines'. Please confirm you have submitted this list.	n.a.
17. For tumor marker prognostic studies, we recommend that you follow the REMARK reporting guidelines (see link list at top right). See author guidelines, under 'Reporting Guidelines'. Please confirm you have followed these guidelines.	n.a.

F- Data Accessibility

18: Provide a "Data Availability" section at the end of the Materials & Methods, listing the accession codes for data generated in this study and deposited in a public database (e.g. RNA-Seq data: Gene Expression Omnibus GSE39462, Proteomics data: PRIDE PXD000208 etc.) Please refer to our author guidelines for 'Data Deposition'.<br><br>Data deposition in a public repository is mandatory for:<br>a. Protein, DNA and RNA sequences<br>b. Macromolecular structures<br>c. Crystallographic data for small molecules<br>d. Functional genomics data<br>e. Proteomics and molecular interactions	Bulk mRNA sequencing data is deposited at GEO under GSE158775 and publicly available.
19. Deposition is strongly recommended for any datasets that are central and integral to the study; please consider the journal's data policy. If no structured public repository exists for a given data type, we encourage the provision of datasets in the manuscript as a Supplementary Document (see author guidelines under 'Expanded View' or in unstructured repositories such as Dryad (see link list at top right) or Figshare (see link list at top right).	Normalised counts of bulk mRNA sequencing was added to the manuscript
20. Access to human clinical and genomic datasets should be provided with as few restrictions as possible while respecting ethical obligations to the patients and relevant medical and legal issues. If practically possible and compatible with the individual consent agreement used in the study, such data should be deposited in one of the major public access-controlled repositories such as dbGAP (see link list at top right) or EGA (see link list at top right).	n.a.
21. Computational models that are central and integral to a study should be shared without restrictions and provided in a machine-readable form. The relevant accession numbers or links should be provided. When possible, standardized format (SBML, CellML) should be used instead of scripts (e.g. MATLAB). Authors are strongly encouraged to follow the MIRIAM guidelines (see link list at top right) and deposit their model in a public database such as Biomodels (see link list at top right) or JWS Online (see link list at top right). If computer source code is provided with the paper, it should be deposited in a public repository or included in supplementary information.	n.a.

G- Dual use research of concern

22. Could your study fall under dual use research restrictions? Please check biosecurity documents (see link list at top right) and list of select agents and toxins (APHIS/CDC) (see link list at top right). According to our biosecurity guidelines, provide a statement only if it could.	No
